# Prolyl Hydroxylase Inhibitor-Mediated HIF Activation Drives Transcriptional Reprogramming in Retinal Pigment Epithelium: Relevance to Chronic Kidney Disease

**DOI:** 10.3390/cells14141121

**Published:** 2025-07-21

**Authors:** Tamás Gáll, Dávid Pethő, Annamária Nagy, Szilárd Póliska, György Balla, József Balla

**Affiliations:** 1 Department of Internal Medicine, Division of Nephrology, Faculty of Medicine, University of Debrecen, H-4032 Debrecen, Hungary; 2 Kálmán Laki Doctoral School, University of Debrecen, H-4032 Debrecen, Hungary; 3 HUN-REN–UD Vascular Biology and Myocardium Pathophysiology Research Group, Hungarian Academy of Sciences, University of Debrecen, H-4032 Debrecen, Hungary; 4 Genomic Medicine and Bioinformatic Core Facility, Department of Biochemistry and Molecular Biology, Faculty of Medicine, University of Debrecen, H-4032 Debrecen, Hungary; poliska@med.unideb.hu; 5 Department of Pediatrics, Faculty of Medicine, University of Debrecen, H-4032 Debrecen, Hungary

**Keywords:** chronic kidney disease, hypoxia, HIF-prolyl-hydroxylase inhibitor, VEGFA, angiogenesis, glycolytic response, oxidative stress, hyperglycemia

## Abstract

Chronic kidney disease (CKD)-associated anemia is a global health concern and is linked to vascular and ocular complications. Hypoxia-inducible factor (HIF) stabilizers, or HIF prolyl hydroxylase inhibitors (PHIs), are promising candidates for the treatment of CKD-associated anemia. Since hypoxia and angiogenesis are involved in eye diseases, this study examined the effects of HIF-PHIs on metabolism and gene expression in retinal pigment epithelium (RPE) cells. Results revealed that PHIs differentially induced angiogenic (VEGFA, ANG) and glycolytic (PDK1, GLUT1) gene expression, with Roxadustat causing the strongest transcriptional changes. However, Roxadustat-induced angiogenic signals did not promote endothelial tube formation. Moreover, it did not induce oxidative stress, inflammation, or significant antioxidant gene responses in ARPE-19 cells. Roxadustat also reduced the inflammatory cytokine response to tumor necrosis factor-α, including IL-6, IL-8, and MCP-1, and did not exacerbate VEGF expression under high-glucose conditions. Overall, Roxadustat triggered complex gene expression changes without promoting inflammation or oxidative stress in RPE cells. Despite these findings, ophthalmologic monitoring is advised during PHI treatment in CKD patients receiving HIF-PHIs.

## 1. Introduction

Anemia is a common feature of chronic kidney disease (CKD) and is a major public health concern worldwide due to its impact on quality of life, as well as its association with increased risk of cardiovascular disease and mortality [1,2,3].

Evidence proves that there is a strong association between the severity of retinopathy and renal function in CKD, with a more pronounced association in patients previously diagnosed with diabetes mellitus [4]. An association between retinal and kidney disease has also been demonstrated by other studies [5,6]. CKD is also associated with diabetic retinopathy (DR) [7]. Several studies have shown a close association between nephropathy and retinopathy in patients with diabetes mellitus [5]. The Chronic Renal Insufficiency Cohort (CRIC) study demonstrated a strong association between severity of retinopathy and level of renal function after adjustment for traditional and non-traditional risk factors for chronic kidney disease, suggesting that retinovascular pathology reflects renal disease [4]. The risk of age-related macular degeneration (AMD) is increased in CKD [8,9].

An effective strategy for the treatment of CKD-associated anemia is the use of pharmacological inhibitors targeting hypoxia-inducible factor (HIF) prolyl-4-hydroxylase domain (PHD) proteins. Under normoxia, PHDs hydroxylate HIFs, promoting their proteosomal degradation, which inhibits the activation of HIF-responsive genes such as vascular endothelial growth factor A (VEGF or VEGF A) and erythropoietin (Epo) [10]. Pharmacological inactivation of PHD enzymes with HIF-prolyl-hydroxilase inhibitors (HIF-PHIs) stimulates endogenous EPO production in both animals and humans [11,12]. Thus, HIF-PHIs (DMOG, Molidustat, Roxadustat, Enarodustat, Daprodustat, and Vadadustat) represent promising alternatives to erythropoiesis-stimulating agents for the treatment of anemia in CKD [13,14].

Pathologic angiogenesis is involved in DR, AMD, and ROP, which are the leading causes of blindness worldwide [15,16,17]. Retinal angiogenesis is closely associated with increasing levels of HIF-1α in DR [18], AMD [19], and ROP [20]. VEGF expression and angiogenesis play a key role in the pathogenesis of DR [21], AMD [22], and ROP [23,24]. CKD and diabetes mellitus frequently co-occur in middle-aged patients, exerting a synergistic detrimental effect on microvascular complications. Among these, a significant concern is the disruption of the blood–retinal barrier, particularly at the level of the retinal pigment epithelium and the retinal endothelium [25]. Hypoxia-inducible gene activators demonstrate efficacy in stimulating red blood cell production comparable to that of erythropoietin therapy. These agents effectively increase and stabilize hemoglobin concentrations and are generally well tolerated by patients with chronic kidney disease (CKD) due to their oral route of administration. However, long-term clinical trials are necessary to fully assess their potential cardiovascular, tumorigenic, and retinopathic risks [26]. CKD and diabetes are frequently combined in middle age patients resulting in a synergistic negative effect on microvascular complications, among them the breakdown of the blood–retinal barrier at the location of retinal pigment epithelial cells and retinal endothelium [25]. Globally, patients with renal failure—particularly those with comorbid diabetes—require effective and safe therapeutic strategies to manage anemia, ensuring that such interventions do not exacerbate, and ideally help to avoid, the progression of proliferative retinopathy [27].

Retinal pigment epithelial cells (RPEs) form the outer blood–retina barrier (BRB), maintain retinal and choroidal homeostasis, produce the high levels of proangiogenic factors, and play a major role in retinal and choroidal neovascularization [28]. RPEs play an important role in the pathology of both DR [29] and AMD [30].

The widely accepted therapeutical indication of PHIs is the kidney-related anemia. Compared to erythropoietin-stimulating agents (ESAs), HIF-PHIs were non-inferior to ESAs in maintaining serum Hb levels in CKD patients with almost similar adverse event profiles [31]. Furthermore, this study also mentions that small sample sizes and short duration periods of the analyzed studies make it difficult to establish the long-term efficacy and safety of HIF-PHI over ESA in the treatment of renal anemia [31]. However, several HIF-PHIs (Roxadustat and DMOG) have been shown to reach retinal tissue, raising concerns that HIF-PHIs may have adverse effects in the retina. In normoxic mice, VEGF is upregulated in a time- and dose-dependent manner in the retinas of mice treated systemically with the HIF activator DMOG [32]. Others have found a two-fold increase in HIF-1α protein levels in response to Roxadustat in the retina in a murine model of ROP [33]. A recent study on mice has shown that Roxadustat induces metabolic dysfunction and neurodegeneration in the retina, which could be potential adverse effects of the prolonged systemic use of HIF-PHIs in certain diseases, such as CKD [34].

CKD-associated anemia is linked to systemic and ocular vascular complications. As HIF stabilizers emerge as treatments for this condition, it is crucial to understand their effects beyond erythropoiesis. Given the central role of hypoxia and angiogenesis in retinal disease, this study investigates how HIF-PHIs influence gene expression and metabolism in RPE cells, which are vital for retinal integrity and function.

In our study, we tested the effects of six clinically relevant small-molecule HIF-PHIs (DMOG, Molidustat, Roxadustat, Enarodustat, Vadadustat, and Daprodustat) on adult retinal pigment epithelial cell-19 (ARPE-19) with a special emphasis on angiogenesis and metabolic alterations, providing insight into the effects of HIF-PHIs in neovascular retinopathies such as DR and AMD.

## 2. Materials and Methods

### 2.1. Reagents

Unless otherwise stated, we obtained all reagents from Sigma-Aldrich (St. Louis, MO, USA). DMOG, Molidustat, Roxadustat, Enarodustat, Daprodustat, and Vadadustat were purchased from Cayman Chemical (Ann Arbor, MI, USA) and dissolved in DMSO. Recombinant human tumor necrosis factor-α (TNF-α) protein was obtained from RD Systems (Abingdon, UK).

### 2.2. Cell Culture

ARPE-19 human retinal pigment epithelial cells (Cat. No. CRL-2302, ATCC, Manassas, VA, USA) were cultured in a 1:1 mixture of Dulbecco’s Modified Eagle Medium (DMEM) and F-12 Medium (ATCC, Manassas, VA, USA), supplemented with 10% fetal bovine serum (FBS) and 1% antibiotic–antimycotic solution, and maintained up to passage 4. From passage 4, cells were maintained in DMEM (low glucose) (Biosera, Cholet, France) supplemented with 5% FBS and antibiotics/antimycotics (Thermo Fisher Scientific, Waltham, MA, USA), (culture medium, CM). Cells between passage 5 and 8 were used for the experiments. Cells were treated with different HIF-PHIs (5, 10, 25 μM) for 16 h or 24 h in a CO_2_ (5%) incubator. For the detection of NF-κB phosphorylation in response to Roxadustat and TNF-α, cells were incubated for 16 h with Roxadustat as described above. Then, cells were washed and exposed to TNF-α (1 or 10 ng/mL) for 10 min in serum-free medium. For the detection of IL-6, IL-8 and MCP-1, cells were incubated for 16 h with Roxadustat as described above. Then, cells were washed and exposed to TNF-α (10 ng/mL) for 24 h in serum-free medium in the presence of Roxadustat (10 μM).

To create a high-glucose condition, DMEM with 5.5 mM glucose, 5% FBS and 1% PSA was supplemented with 19.5 mM glucose (25 mM total glucose) or mannitol (5.5 mM glucose and 19.5 mM mannitol) as an osmotic control. Cells were cultured in these media for 24 h, then exposed to Roxadustat (5 μM) in the appropriate medium for 24 h.

### 2.3. RNA Isolation and Real-Time Quantitative Polymerase Chain Reaction

Total RNA was extracted 16 h post-treatment using TriReagent (Zymo Research, Irvine, CA, USA), followed by reverse transcription into cDNA using the High-Capacity cDNA Reverse Transcription Kit (Applied Biosystems, Foster City, CA, USA). VEGFA (Hs00900055_m1), GLUT1 (Hs00892681_m1), PDK1 (Hs01561847_m1), ANG (Hs04195574_sh), and RNA45S5 (Hs05627131_gH) mRNA expressions were determined by TaqMan Gene Expression Assays (Thermo Fisher Scientific, Waltham, MA, USA). Relative mRNA expression levels were determined using the ΔΔCt method, with RNA45S5 serving as the endogenous reference gene.

### 2.4. Cell Lysis and Western Blot

Cells were lysed using RIPA buffer supplemented with protease and phosphatase inhibitors. Protein concentrations were quantified using the bicinchoninic acid (BCA) assay (Pierce BCA Protein Assay Kit, Thermo Fisher Scientific, Waltham, MA, USA). Equal amounts of protein were separated by SDS-PAGE on Tris–glycine gels, followed by transfer onto 0.22 µm nitrocellulose membranes (GE Healthcare, Chicago, IL, USA) [24], blocked and probed according to the manufacturer’s guide with primary antibodies against Phospho-NF-κB p65 (Ser536) (Cat. No. #3033, Cell Signaling Technologies, Danvers, MA, USA), stripped and reprobed with primary antibodies against NF-κB p65 (Cat. No. #8242, Cell Signaling Technologies, Danvers, MA, USA) and β-Actin (Rabbit (Cat. No. #4970, Cell Signaling Technologies, Danvers, MA, USA), incubated with Anti-rabbit IgG, HRP-linked Antibody (Cell Signaling Technology, Danvers, MA, USA). The antigen–antibody complexes were visualized using the WesternBright Quantum HRP substrate (Advansta, Menlo Park, CA, USA). Immunoblot images were captured with the iBright FL1500 imaging system (Thermo Fisher Scientific, Waltham, MA, USA).

### 2.5. Human VEGF, IL-6, IL-8, and MCP-1 ELISA

Supernatants from ARPE-19 cells exposed to HIF-PHIs were collected 24 h after the treatments and measured using Human VEGF Quantikine ELISA assay (R&D Systems, Abingdon, UK), while IL-6 or IL-8 or MCP-1 levels in response to Roxadustat and TNF-α were collected 24 h after the treatments and analyzed using IL-6 or IL-8 or MCP-1 Quantikine ELISA assay (R&D Systems, Abingdon, UK). Protein expressions were normalized to total protein content.

### 2.6. RNA-Seq Method

RNA isolations were performed as described earlier [35]. Global transcriptome profiling was conducted through mRNA sequencing using the Illumina platform. RNA-Seq libraries were prepared from total RNA using Ultra II RNA Sample Prep kit (New England BioLabs, Ipswich, MA, USA) according to the manufacturer’s protocol. Sequencing runs were executed on the Illumina NextSeq 500 instrument (Illumina, San Diego, CA, USA) using single-end 75 cycles sequencing. Single-read 75 bp long sequencing was performed, the base calling accuracy Q30 was >93%. The average read number was 25.1 million reads per samples; the read numbers varied between 21.5 and 28.9 million. Raw sequencing reads were aligned to the human reference genome version GRCh38, the alignment percentage was >95%. Aligned sequencing data have been deposited into the NCBI SRA database under accession no. PRJNA1092586.

### 2.7. RNA-Seq Data Analysis

RNA-Seq data analysis was performed as described earlier [24]. Briefly, Raw sequencing reads in FASTQ format were aligned to the human reference genome (GRCh38) using the HISAT2 aligner, resulting in BAM file generation. Subsequent analysis was performed with the Strand NGS platform www.strand-ngs.com (accessed on 9 January 2024). The BAM files were imported into the software, and data normalization was carried out using the DESeq algorithm. Differential gene expression between conditions was assessed using a moderated *t*-test, with a *p*-value threshold of <0.05 considered statistically significant [24].

### 2.8. Pathway Analyses

Cytoscape v3.4, along with the ClueGO v2.3.5 plugin, was utilized to identify significantly enriched Gene Ontology (GO) terms. A two-sided hypergeometric test was conducted to compare the list of differentially expressed genes against the GO Biological Process database.

### 2.9. Confocal Microscopy

ARPE-19 cells cultured on coverslips were treated as described above. For GLUT1 staining, cells were fixed with 4% paraformaldehyde solution. Samples were permeabilized and blocked with 5% normal goat serum/2% BSA/0.1% saponin for 60 min. Primary antibody against GLUT1 (Cat. No. ab115730, abcam, Cambridge, UK, dilution 1:500) was diluted in 1% BSA/0.1% saponin in PBS and incubated overnight at 4 °C, followed by an incubation with goat anti-rabbit IgG conjugated to Alexa Fluor 488 0.1% BSA/0.1% saponin in PBS (Thermo Fisher Scientific, Waltham, MA, USA). For PDK1 staining, cells were fixed at room temperature with ethanol cooled to −20 °C, permeabilized with 0.2% Triton X-100 in PBS, blocked with 5% normal goat serum/2% BSA and incubated with primary antibody against PDK1 (Proteintech, Manchester, UK dilution 1:400). Primary antibody against HIF-2α (dilution 1:100; Cat. No. #71565, Cell Signaling Technology, Danvers, MA, USA) was applied in antibody dilution buffer (1% BSA/0.3% Triton X-100 in PBS) and incubated overnight at 4 °C. Primary antibody against HIF-1α (dilution 1:400, Cat. no. #36169, Cell Signaling Technology, Danvers, MA, USA) and VEGF (dilution 1:100, MA5-13182, Thermo Fisher Scientific, Waltham, MA, USA) were diluted in 1% BSA and incubated overnight at 4 °C. Samples were then incubated then with secondary antibodies conjugated to Alexa Fluor 488 or/and 647 (Thermo Fisher Scientific, Waltham, MA, USA). For the detection of nuclear factor kappa B (NFκB), cells were exposed to Roxadustat (10 μM) for 24 h. Primary antibody against NFκB (dilution 1:800; Cat. No. #8242, Cell Signaling Tech-nology, Danvers, MA, USA) was applied in antibody dilution buffer (1% BSA/0.3% Triton X-100 in PBS) and kept at 4 °C overnight. Samples were then incubated then with secondary antibodies conjugated to Alexa Fluor 488 (Thermo Fisher Scientific, Waltham, MA, USA).

Nuclei were stained with Hoechst. Immunofluorescent analysis was performed with lightning super-resolution microscopy using Leica Application Software X v1.4.7.28982 (Leica, Mannheim, Germany).

### 2.10. Endothelial Cell Tube Formation Assay

The formation of endothelial cell tube networks was analyzed using an Angiogenesis Starter Kit (GIBCO, Thermo Fisher Scientific, Waltham, MA, USA). To produce conditioned culture media, ARPE-19 cells were exposed to Roxadustat (25 μM) for 16 h as described above and cultured in Medium 200 supplemented with 2% FBS for 16 h. Then, supernatants were collected. Endothelial tube formation assay (ln Vitro Angiogenesis) was performed as recommended by the Angiogenesis Starter Kit. Human umbilical vein endothelial cells (HUVECs) were cultured in Medium 200 supplemented with Large VesseI Endothelial Supplement (LVES). A 35 mm glass-bottom dish (Ibidi, Gräfelfing, Germany) was coated with Geltrex LDEV-Free Reduced Growth Factor Basement Membrane Matrix according to the manufacturer’s guide. HUVECs were trypsinized and resuspended in Medium 200 containing 2% FBS without LVES. Then, 75,000 cells (in a maximal final volume of 110 μL) in Medium supplemented with 2% FBS without LVES were plated in 1 mL of conditioned medium from ARPE-19 cells and incubated for 16 h. For a positive control of tube formation, HUVECs were plated in Medium 200 supplemented with LVES. HUVECs plated in Medium 200 with 2% FBS without LVES serving as negative controls. Tube formation was observed using the Leica DMi1 microscope. Tube formation analysis was performed with Fiji software v. 1.54p [36] using the Angiogenesis Analyzer plugin [37].

### 2.11. Oxidative Stress Detection

Cells were incubated with 5 µM CellROX Green Reagent (Thermo Fisher Scientific, Waltham, MA, USA) by adding the dye directly to the complete growth medium and maintaining the culture at 37 °C for 30 min. Following incubation, cells were washed three times with phosphate-buffered saline (PBS) and subsequently fixed using a 3.7% paraformaldehyde solution. Nuclear staining was performed using Hoechst dye, and the samples were immediately examined using confocal microscopy (Leica, Mannheim, Germany).

To detect mitochondrial superoxide production, cells were exposed to Roxadustat (10 μM) for 16 h or Menadione (50 μM) for 1 h. The cells were then stained with 5 nM MitoSOX Red for 30 min (Thermo Fisher Scientific, Waltham, MA, USA). Cells were then washed three times with phosphate-buffered saline, and samples were immediately analyzed using confocal microscopy (Leica, Mannheim, Germany) in FluoroBright DMEM (Thermo Fisher Scientific, Waltham, MA, USA).

### 2.12. Statistical Analysis

All data were obtained after at least three independent experiments. Statistical analysis was carried out by one-way ANOVA test followed by Bonferroni correction or *t*-test using GraphPad Prism 5.0 software (GraphPad Software, Boston, MA, USA). A value of *p* < 0.05 was considered statistically significant (* *p* < 0.05, ** *p* < 0.01, *** *p* < 0.001). Data are represented as mean value ± SEM from at least three independent experiments. For RNA-Seq data analysis, a moderated *t*-test was used to determine differentially expressed genes between conditions, *p* < 0.05 was considered a significant difference.

## 3. Results

### 3.1. HIF-PHIs Differentially Induced VEGF, ANG, PDK1, and GLUT1 Expression in ARPE-19 Cells

**Figure 1:**
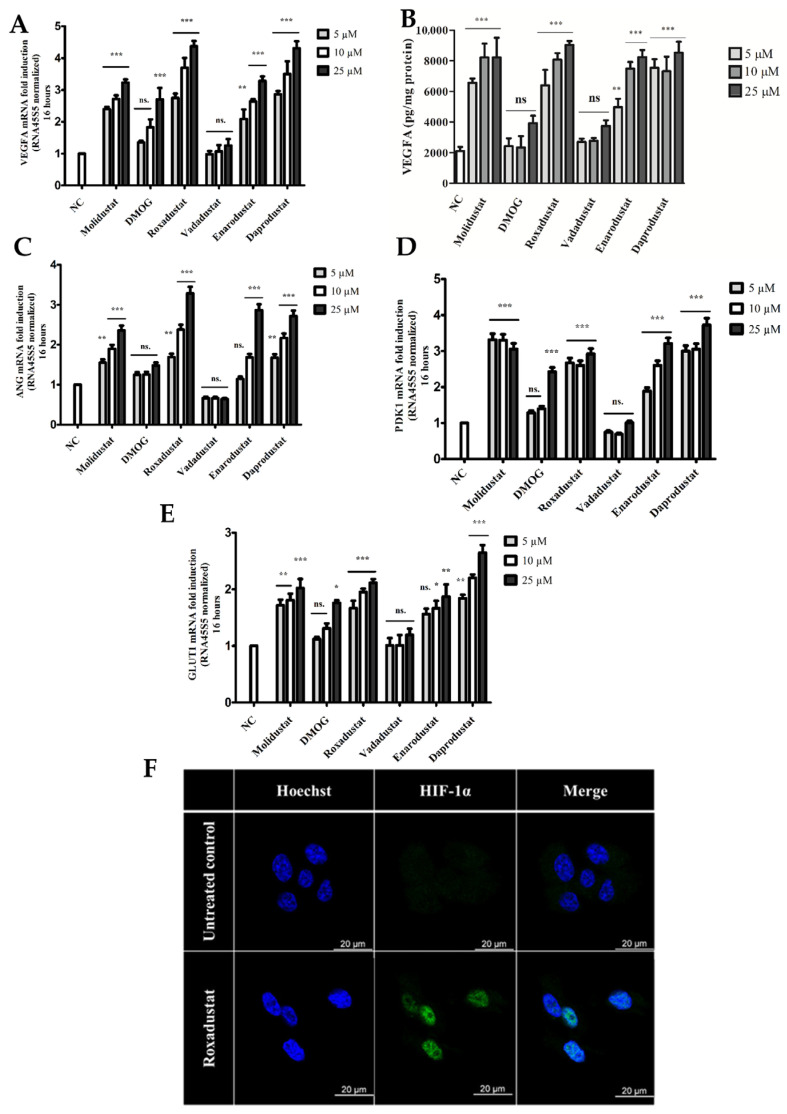
Differential induction of VEGFA, ANG, PDK1, and GLUT1 expression by HIF-PHIs in ARPE-19 cells. Human retinal pigment epithelial cells (ARPE-19) were treated with varying concentrations (5, 10, and 25 μM) of Molidustat, DMOG, Roxadustat, Vadadustat, Enarodustat, and Daprodustat. NC: non-treated control. (**A**) VEGFA mRNA levels were analyzed by quantitative real-time PCR (qRT-PCR) after 16 h of treatment. (**B**) VEGFA protein levels in cell culture supernatants were measured by ELISA after 24 h. (**C**–**E**) mRNA levels of (**C**) Angiopoietin (ANG), (**D**) Pyruvate Dehydrogenase Kinase 1 (PDK1), and (**E**) Glucose Transporter 1 (GLUT1) were measured by qRT-PCR after 16 h of treatment. (**F**) HIF-1α nuclear translocation was assessed in ARPE-19 cells treated with Roxadustat (10 μM) for 3 h. Nuclei were counterstained with Hoechst.

Since the effects of PHIs occur via HIF-1α-mediated regulation, we compared the activities of different PHIs in human retinal pigment epithelial cells focusing on hypoxic gene expressions. The effect of varying concentrations (5, 10, 25 μM) of HIF-PHIs (Molidustat, DMOG, Roxadustat, Vadadustat, Enarodustat, and Daprodustat) on VEGF expression in ARPE-19 cells was evaluated using RT-qPCR and ELISA. Significant VEGF mRNA and protein expression was observed with Molidustat, Roxadustat, Enarodustat, and Daprodustat at all applied concentrations in a dose-dependent manner (Figure 1A,B). The levels of VEGF protein expression were similar for all HIF-PHIs except for Enarodustat at 5 μM, which resulted in lower VEGF protein expression than Molidustat, Roxadustat, and Daprodustat at the same concentration. Vadadustat did not significantly induce VEGF expression at either the RNA or protein level (Figure 1A,B). DMOG induced significant VEGF mRNA expression only at the highest concentration (25 μM), but this was not statistically significant at the protein level (Figure 1A,B). A similar trend was noted for the expression of Angiogenin (ANG) (Figure 1C). ANG mRNA expression was not significantly induced by DMOG (5, 10, 25 μM), Vadadustat (5, 10, 25 μM), and the lowest Enarodustat concentration (5 μM), while higher Enarodustat concentrations as well as Molidustat, Roxadustat, and Daprodustat significantly induced ANG expression at all doses (Figure 1C).

**Figure 2:**
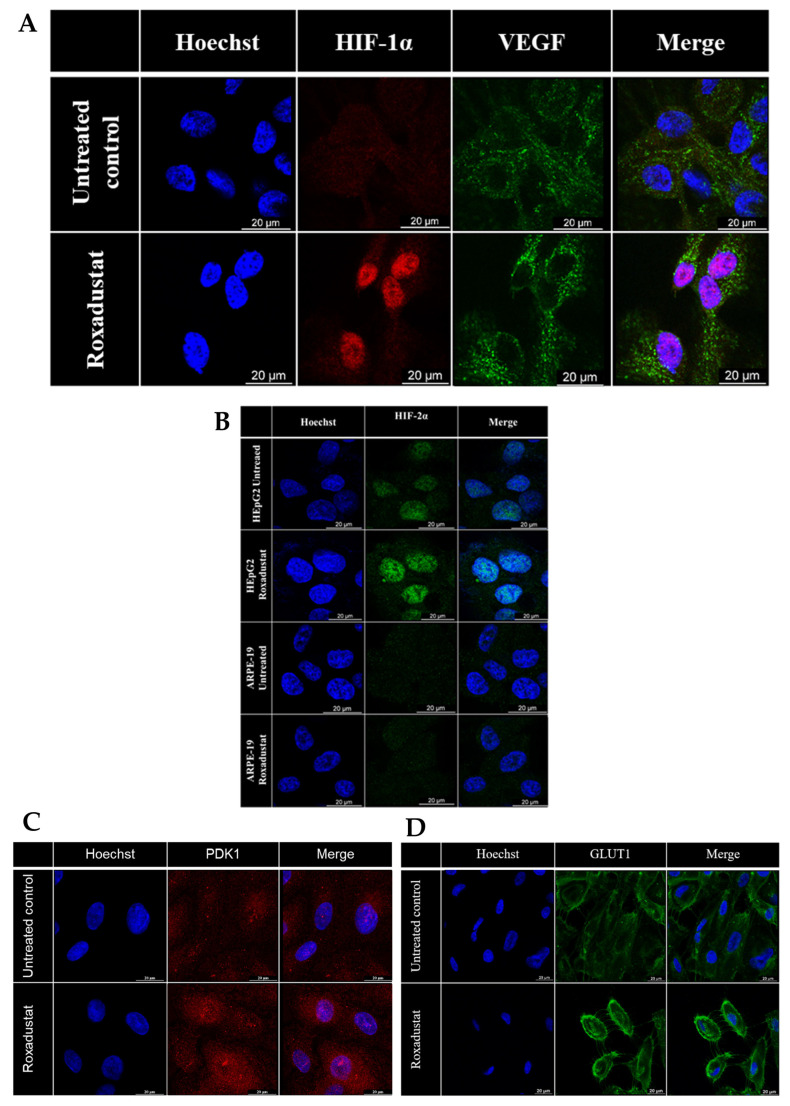
Expression of VEGF, HIF-1α, HIF-2α, PDK1, and GLUT1 in ARPE-19 cells in response to Roxadustat. (**A**) Immunofluorescence detection of HIF-1α and VEGF in ARPE-19 cells treated with Roxadustat (10 μM) for 16 h. (**B**) Detection of HIF-2α in ARPE-19 and HEpG2 cells after treatment with Roxadustat (10 μM, 16 h). (**C**,**D**) Detection of (**C**) PDK1 and (**D**) GLUT1 in ARPE-19 cells following treatment with Roxadustat (10 μM, 16 h). Nuclei were counterstained with Hoechst.

The genes regulated by HIF-1α play a crucial role in the pathology of ocular diseases [18,19,20,38]. Therefore, we investigated the impact of various HIF-PHIs on the expression of two HIF-regulated proteins, Pyruvate dehydrogenase kinase 1 (PDK1) and Glucose transporter 1 (GLUT1) (Figure 1D,E). No changes in PDK1 expression were observed after treatment with Vadadustat at any concentration compared to the control. DMOG induced a significant increase in PDK1 expression only at the highest concentration (25 μM) compared to the control (Figure 1D). Molidustat, Roxadustat, Enarodustat, and Daprodustat significantly induced PDK1 gene expression at all concentrations, with the lowest expression observed in the case of 5 μM of Enarodustat (Figure 1D). Similar to PDK1, Vadadustat did not significantly induce GLUT1 expression, while DMOG triggered GLUT1 expression only at the highest concentration (25 μM) (Figure 1E). Molidustat, Roxadustat, Enarodustat, and Daprodustat significantly induced GLUT1 gene expression at all concentrations except for the lowest (5 μM) dose of Enarodustat (Figure 1E). Immunofluorescent analysis of ARPE-19 cells treated with Roxadustat (10 μM) revealed the nuclear translocation of HIF-1α as early as 3 h post-treatment (Figure 1F). Subsequently, we evaluated the dynamics of HIF-1α nuclear translocation. The results demonstrated that HIF-1α exhibited nuclear translocation in conjunction with VEGFA induction in Roxadustat (10 μM)-treated cells after 16 h (Figure 2A). In contrast, Roxadustat-treated ARPE-19 cells exhibited no HIF-2α nuclear translocation in comparison to the control HEpG2 cells (Figure 2B). Immunofluorescent protein staining also confirmed increased expression of both PDK1 (Figure 2C) and GLUT1 (Figure 2D) in the protein levels of in a selected HIF-PHI, Roxadustat (10 μM) after 16 h.

These data indicated HIF-PHIs differentially induced VEGF, ANG, PDK1, and GLUT1 in ARPE-19 cells. Furthermore, our findings demonstrated that Roxadustat selectively induced the nuclear translocation of HIF-1α, but not HIF-2α, in these cells.

### 3.2. Transcriptional Responses of ARPE-19 Cells to Roxadustat

**Figure 3:**
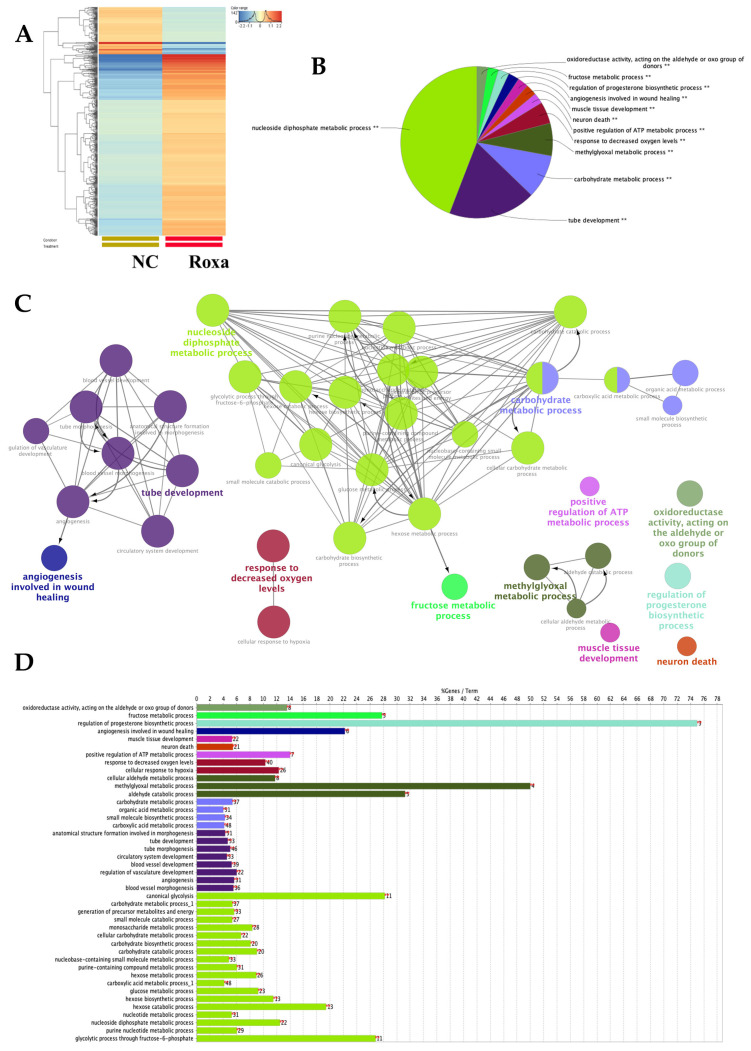
Transcriptomic signatures of ARPE-19 cells in response to Roxadustat exposure. (**A**) Heatmap showing differentially expressed genes in ARPE-19 cells treated with Roxadustat (10 μM, 16 h) compared to non-treated control (NC) cells. ‘Roxa’ refers to Roxadustat-treated samples. The color scale represents relative gene expression levels, with red indicating upregulation and blue indicating downregulation. (**B**) Gene Ontology (GO) enrichment analysis of significantly altered pathways in Roxadustat-treated cells, performed using the ClueGO plugin in Cytoscape. (**C**) Graphical representation of enriched biological processes (BPs); node colors correspond to functional group classifications. (**D**) Percentage of genes associated with the identified GO terms.

To study the gene expression characteristic of one of the most effective drugs in our RPE cell model, we analyzed the transcriptional responses to HIF-PHI Roxadustat. Unbiased high-throughput RNA sequencing (RNA-seq) was performed on ARPE-19 cells exposed to Roxadustat (10 μM for 16 h). This analysis revealed 469 differentially expressed genes (DEGs) that met our inclusion criteria for fold-change expression (>2) in Roxadustat-stimulated ARPE-19s relative to the control (Figure 3A). The control group clearly separated from the Roxadustat group in the heat map (Figure 3A).

Next, we identified DEGs’ function using the Cytoscape ClueGO [39]. This revealed that overrepresented GOterms in Roxadustat-stimulated cells were mainly associated with nucleoside diphosphate metabolic processes, tube development, carbohydrate metabolic processes, methylglyoxal metabolic processes, response to decreased oxygen levels, positive regulation of ATP metabolic processes, neuron death, muscle tissue development, angiogenesis involved in wound healing, regulation of progesterone biosynthetic processes, fructose metabolic processes, and oxidoreductase activity, acting on the aldehyde or oxo group of donors (Figure 3B,C). Details of each enriched ClueGO group are shown in Figure 3D.

**Figure 4:**
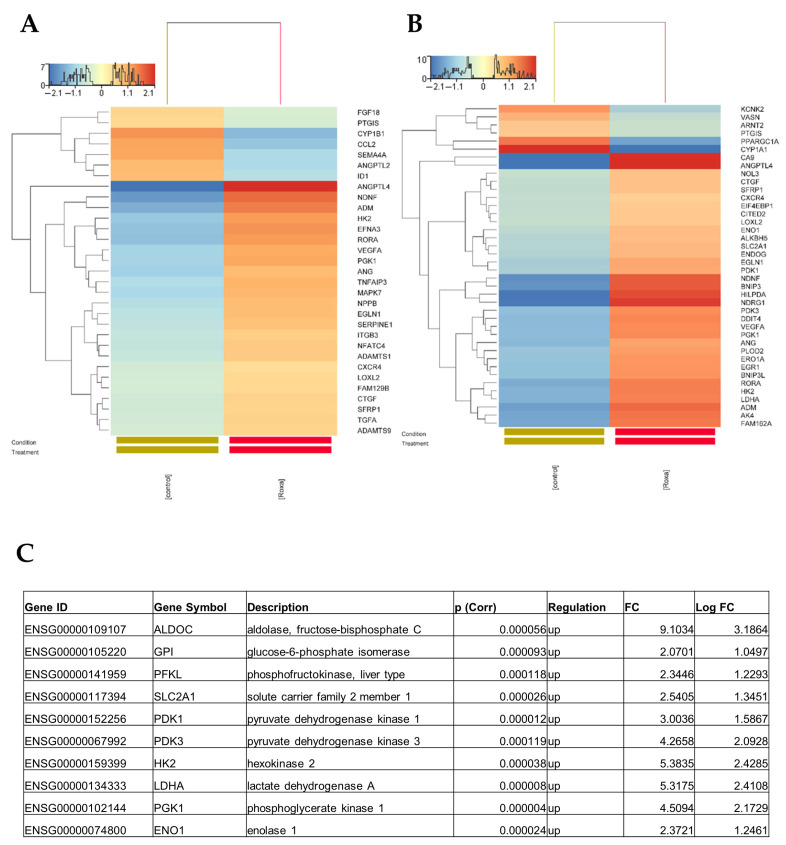
Transcriptomic signatures of ARPE-19 cells following Roxadustat exposure. (**A**) Differential expression of genes annotated as positive regulators of angiogenesis in ARPE-19 cells treated with Roxadustat (10 μM, 16 h) compared to non-treated controls. (**B**) Genes associated with the cellular response to decreased oxygen levels. (**C**) Expression of glycolysis-related genes in Roxadustat-stimulated ARPE-19 cells versus controls. *p* (adjusted) < 0.05. FC: fold change.

Given that angiogenesis [15,16,17], hypoxic response [18,19,40], and increased glycolysis [41] are involved in retinopathies, we next examined the expression of these genes with special interest (Figure 4A–C).

Among genes annotated to angiogenesis, 31 genes (5.64 per cent of annotated to angiogenesis) showed significant change. Out of these, 24 were significantly increased (ADAMTS1, ADAMTS9, ADM, ANG, ANGPTL4, CTGF, CXCR4, EFNA3, EGLN1, HK2, ITGB3, LOXL2, MAPK7, NDNF, NFATC4, FAM129B, NPPB, PGK1, RORA, SERPINE1, SFRP1, TGFA, TNFAIP3, VEGFA), while 7 were significantly decreased (FGF18, PTGIS, CYP1B1, CCL2, SEMA4A, ANGPTL2, ID1) in response to Roxadustat (10 μM) (Figure 4A). Among genes annotated to decreased oxygen levels, 40 genes (10.31 per cent annotated to this GOterm) showed significant change. Out of these, 34 were significantly induced (ADM, AK4, ALKBH5, ANG, ANGPTL4, BNIP3, BNIP3L, CA9, CTGF, CITED2, CXCR4, DDIT4, EGLN1, EGR1, EIF4EBP1, ENDOG, ENO1, ERO1A, FAM162A, HILPDA, HK2, LDHA, LOXL2, NDNF, NDRG1, NOL3, PDK1, PDK3, PGK1, PLOD2, RORA, SFRP1, SLC2A1, VEGFA), while 6 were significantly decreased (KCNK2, VASN, ARNT2, PTGIS, PPARGC1A, CYP1A1) in response to Roxadustat (10 μM) (Figure 4B). List of gene abbreviations are depicted in the Supporting Information as Supplementary Table S1.

To evaluate the effect of HIF-1α activation on glycolysis in ARPE-19 cells after Roxadustat treatment, we analyzed the expression ALDOC (Aldolase, fructose-bisphosphate C), GPI (Glucose-6-phosphate isomerase), PFKL (Phosphofructokinase, liver type), GLUT1 (also known SLC2A1, solute carrier family 2 member 1), PDK1, PDK3 (Pyruvate dehydrogenase kinase 3), HK2 (Hexokinase 2), LDHA (Lactate dehydrogenase A), PGK1 (Phosphoglycerate kinase 1), and ENO1 (Enolase 1). We showed that all of these proteins were robustly upregulated (fold-change expression > 2) in the Roxadustat group (Figure 4C).

Together, our results showed that many genes involved in angiogenesis and hypoxic response involving glycolysis were robustly induced by Roxadustat in ARPE-19 cells.

### 3.3. HIF-PHI Roxadustat Does Not Induce Endothelial Tube Formation in HUVEC Cultures

**Figure 5:**
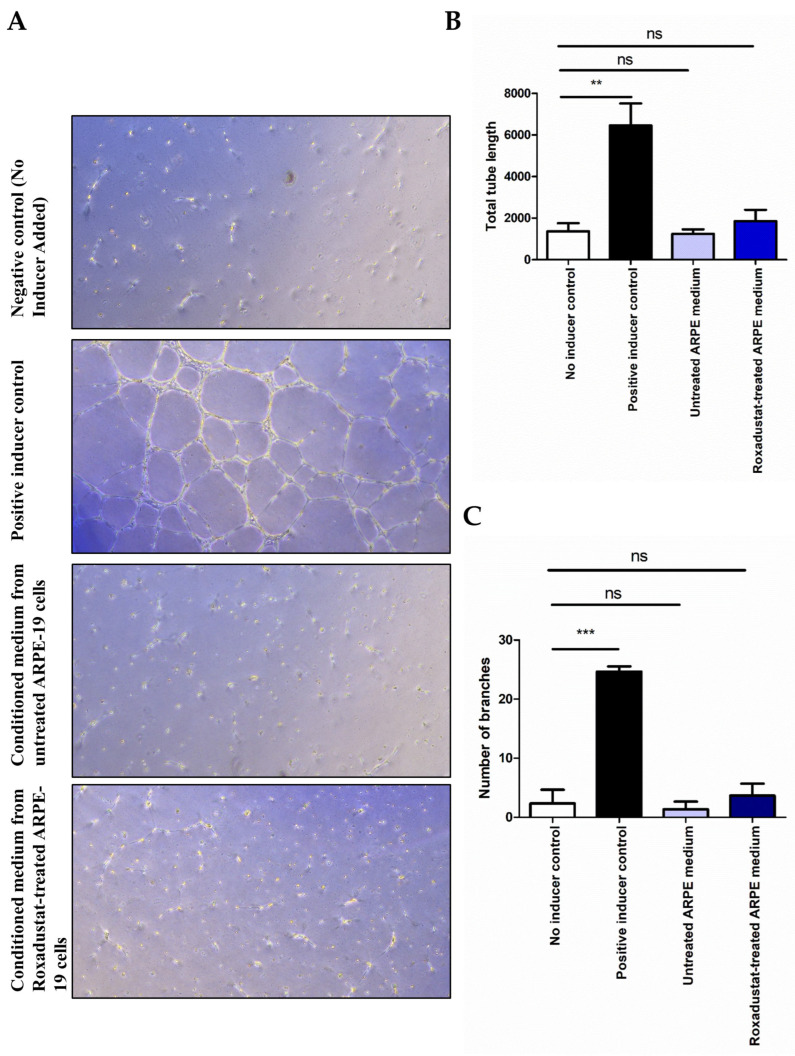
Endothelial cell tube formation assay using ARPE-19 conditioned media. ARPE-19 cells were treated with Roxadustat (10 μM, 16 h), and the resulting cell culture supernatants were collected. Human umbilical vein endothelial cells (HUVECs) were then seeded onto Geltrex™ LDEV-Free Reduced Growth Factor Basement Membrane Matrix and incubated with the conditioned media. For controls, HUVECs were cultured in Medium 200 supplemented with Large Vessel Endothelial Supplement (LVES) as a positive control for tube formation, and in Medium 200 without LVES as a negative control. (**A**) Tube formation was visualized using a Leica DMi1 microscope. (**B**) Total tube length and (**C**) the number of branches were analyzed by Fiji software v. 1.54p with Angiogenesis Analyzer plugin.

To ascertain whether the secretome derived from Roxadustat-treated ARPE-19 cells stimulates endothelial tube formation in HUVEC cultures, a tube formation assay was conducted in accordance with the methodology outlined in the Section 2. The results demonstrated that the conditioned media from ARPE-19 cells treated with Roxadustat (10 μM) for 24 h did not induce significant tube formation in HUVEC cultures on Geltrex LDEV-Free Reduced Growth Factor Basement Membrane Matrix (Figure 5A).

However, HUVEC cultures exposed to the positive inducer control demonstrated notable tube formation (Figure 5A). This was corroborated by assessing the total tube length (Figure 5B) and the number of branches (Figure 5C). These findings indicate that Roxadustat did not augment the angiogenic capacity of the ARPE-19 secretome in an in vitro tube formation assay.

### 3.4. Investigation of Antioxidant Gene Expression and Reactive Oxygen Species (ROS) Generation in ARPE-19 Cells in Response to Roxadustat

**Figure 6:**
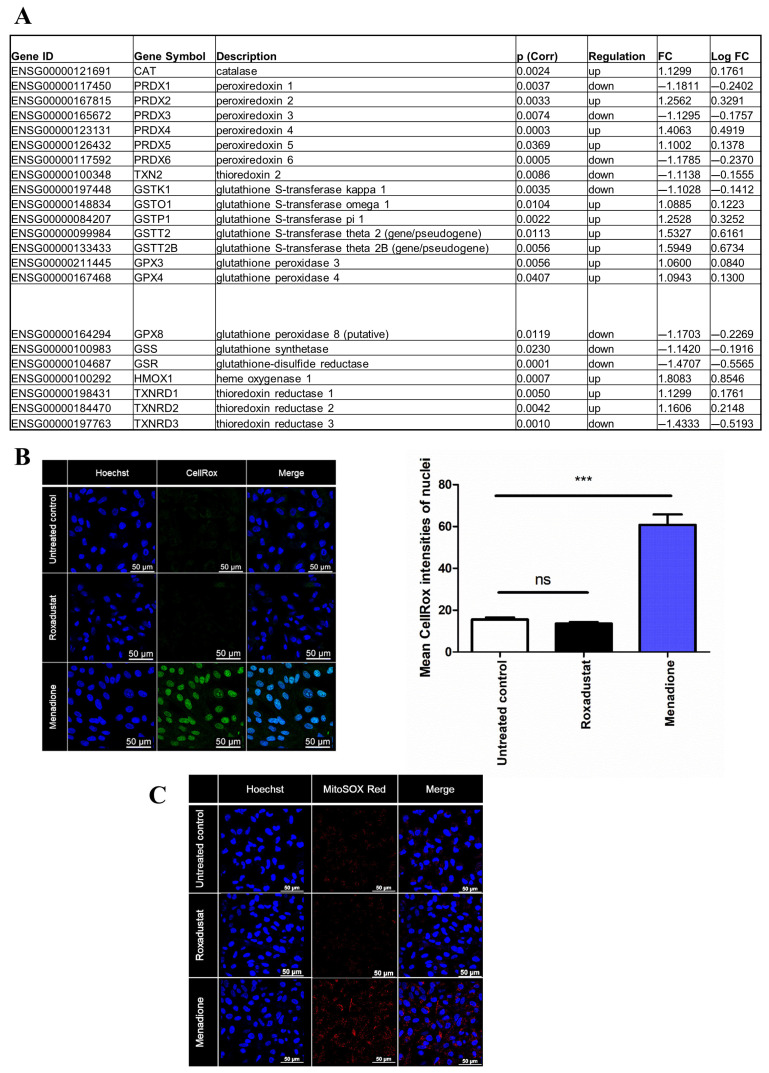
Antioxidant gene expression and reactive oxygen species (ROS) generation in ARPE-19 cells following Roxadustat treatment. (**A**) Expression levels of altered antioxidant genes in ARPE-19 cells treated with Roxadustat (10 μM, 16 h). *p* (corr): adjusted *p*-values; FC: fold change. (**B**) Detection of oxidative stress using CellROX™ dye in ARPE-19 cells treated with Roxadustat. Mean nuclear fluorescence intensities were quantified from 50 cells (N = 50) using LAS X software Version 5.3.0. (**C**) Detection of mitochondrial superoxide production using MitoSOX™ Red dye in ARPE-19 cells after Roxadustat exposure. Nuclei were counterstained with Hoechst.

DEGs involved in antioxidant response which significantly changed (*p* < 0.05) are depicted in Figure 6A.

Importantly, DEGs that met our inclusion criteria for fold-change expression (>2) in Roxadustat-stimulated ARPEs relative to the control did not include any antioxidant genes. Therefore, we set inclusion criteria for fold-change expression to 1. This change resulted in identifying the following up-regulated genes: catalase (CAT), peroxiredoxin 2 (PRDX2), peroxiredoxin 4 (PRDX4), peroxiredoxin 5 (PRDX5), glutathione S-transferase omega 1 (GSTO1), glutathione S-transferase pi 1 (GSTP1), glutathione S-transferase theta 2 (gene/pseudogene) (GSTT2), glutathione S-transferase theta 2B (gene/pseudogene) (GSTT2B), glutathione peroxidase 3 (GPX3), glutathione peroxidase 4 (GPX4), heme oxygenase 1 (HMOX1), thioredoxin reductase 1 (TXNRD1), and thioredoxin reductase 2 (TXNRD2), while downregulated genes involved peroxiredoxin 1 (PRDX1), peroxiredoxin 3 (PRDX3), peroxiredoxin 6 (PRDX6), thioredoxin 2 (TXN2), glutathione S-transferase kappa 1 (GSTK1), glutathione peroxidase 8 (putative) (GPX8), glutathione synthetase (GSS), glutathione-disulfide reductase (GSR), and thioredoxin reductase 3 (TXNRD3) (Figure 6A). Interestingly, the expressions of superoxide dismutase 1 and 2 showed no significant difference between the Roxadustat-treated group and the control group. Given that the majority of antioxidant genes showed low FC values but were significantly induced by Roxadustat, we measured ROS generation using CellRox staining (Figure 6B). This revealed that ROS generated by Roxadustat-treated cells did not differ from ROS generation detected in the control group, while the cells exposed to menadione showed significant ROS generation (Figure 6B). Similar to this, Roxadustat did not induce mitochondrial superoxide production (Figure 6C).

### 3.5. Roxadustat Does Not Induce NFκB Nuclear Tranlocation. Roxadustat Mitigates Inflammatory Response to TNF-α. High Glucose Does Not Aggravate Roxadustat-Induced VEGF Expression in ARPE-19 Cells

**Figure 7:**
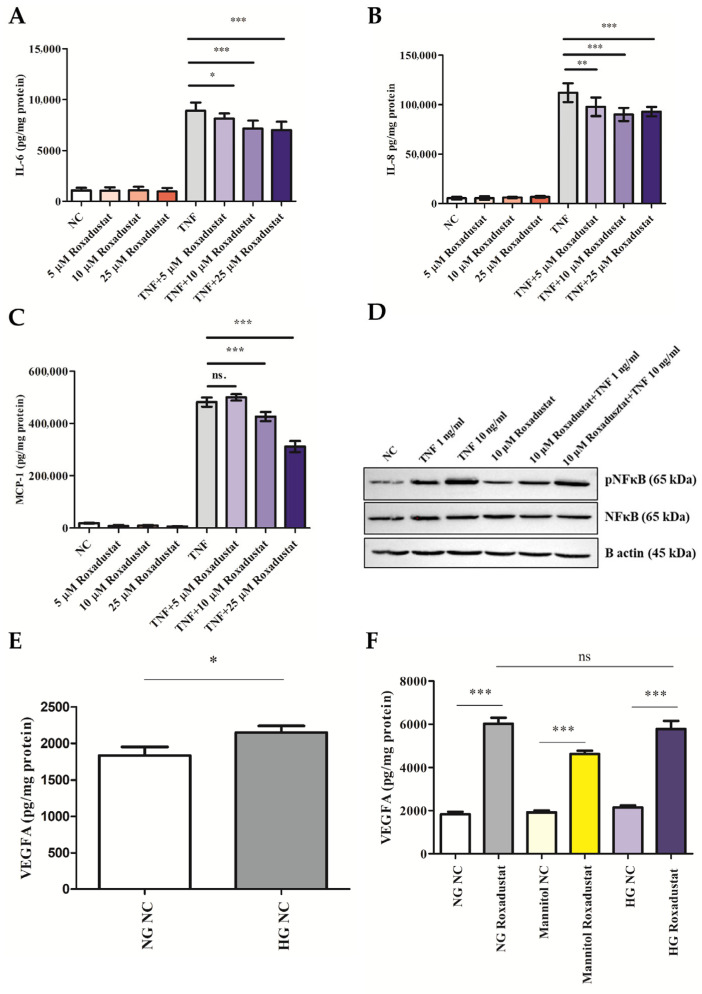
Roxadustat reduces TNF-α-induced inflammation and VEGFA expression is not enhanced by high glucose in ARPE-19 cells. ARPE-19 cells were pretreated with Roxadustat (5, 10, or 25 μM) for 16 h, followed by stimulation with TNF-α (10 ng/mL) for 24 h, in the continued presence or absence of Roxadustat. (**A**–**C**) Protein levels of (**A**) IL-6, (**B**) IL-8, and (**C**) MCP-1 in cell culture supernatants were measured by ELISA. Cytokine levels were normalized to total protein content in cell lysates. (**D**) Detection of NF-κB p65 phosphorylation in ARPE-19 cells pretreated with Roxadustat (10 μM, 16 h), followed by TNF-α stimulation (1 or 10 ng/mL) for 10 min. (**E**) VEGF protein secretion in ARPE-19 cells cultured in normal glucose (5.5 mM, NG) or high glucose (25 mM, HG) conditions. NG NC: normal glucose, untreated control; HG NC: high glucose, untreated control. (**F**) VEGF protein levels in ARPE-19 cells cultured in NG (5.5 mM glucose), HG (25 mM glucose), or osmotic control (5.5 mM glucose + 19.5 mM mannitol), with or without Roxadustat (5 μM, 24 h). NG NC and HG NC indicate normal and high glucose untreated controls, respectively.

First, we analyzed whether Roxadustat triggers the nuclear translocation of NFκB. We showed that Roxadustat (10 μM) did not facilitate the nuclear translocation of NFκB even after 24 h of incubation (Figure S1). In addition, our findings demonstrated that Roxadustat, when administered alone, did not result in an increase in IL-6 secretion in ARPE-19 cells. However, it was observed to significantly attenuate TNF-α-induced IL-6 secretion (Figure 7A). Subsequently, IL-8 secretion was quantified in response to TNF-α alone or in combination with Roxadustat. The results demonstrated that Roxadustat alone did not increase IL-8 secretion. However, similar to IL-6, it significantly decreased TNF-α-induced IL-8 secretion (Figure 7B). Next, we measured TNF-α-driven MCP-1 secretion in the presence or absence of Roxadustat. The results demonstrated that Roxadustat alone did not elevate MCP-1 secretion; however, it markedly reduced MCP-1 secretion induced by TNF-α at the higher concentrations (10 and 25 μM) (Figure 7C). While Roxadustat treatment lowered the TNF-α–induced increase in inflammatory marker (IL-6, IL-8, MCP-1) levels, expression remained markedly elevated compared to untreated controls.

To investigate the anti-inflammatory impact of Roxadustat on TNF-α-induced inflammatory signaling in ARPE-19 cells, we conducted a Western blot analysis to examine the phosphorylation of NF-κB and MAPK signaling pathway component JNK. Our findings demonstrated that TNF-α (1 and 10 ng/mL) markedly enhanced the phosphorylation of NF-κB p65 in a dose-dependent manner, whereas Roxadustat alone exhibited no discernible impact on NF-κB phosphorylation (Figure 7D). Roxadustat was observed to reduce NF-κB phosphorylation in TNF-α-treated ARPE-19 cells (Figure 7D). In conclusion, Roxadustat alone did not increase the phosphorylation of NF-κB p65, but reduced TNF-α-induced NF-κB phosphorylation.

Hyperglycemia is known to aggravate VEGF secretion in ARPE-19 cells [42]. Accordingly, we proceeded to assess the impact of high glucose on Roxadustat-induced VEGF secretion. As illustrated in Figure 7E, high glucose (25 mM glucose) was observed to markedly enhance VEGF secretion in comparison to the normal glucose (5.5 mM) control (Figure 7E). Our results showed that Roxadustat (10 μM) significantly increased VEGF secretion in the normal glucose (5.5 mM), high glucose (25 mM glucose), and in the osmotic control group (19.5 mM mannitol + 5.5 mM glucose) (Figure 7F). It is noteworthy that high glucose did not significantly elevate Roxadustat-induced VEGF secretion compared to the normal glucose group in ARPE-19 cells (Figure 7F).

## 4. Discussion

VEGF expression and angiogenesis play a key role in the pathogenesis of DR [21], AMD [22], and ROP [23]. In the retina, both in normal development and in the context of pathologic hypoxia, VEGF is the dominant mediator of the hypoxia-driven proangiogenic response [43]. It can be postulated that the activation of the HIF pathway may enhance retinal angiogenesis and predispose patients to retinal complications such as neovascularization and hemorrhage. The present study examined the effects of HIF-PHIs in a retinal pigment epithelial cell model, with the objective of elucidating their impact on angiogenesis, oxidative stress, and inflammation.

The present study revealed that the VEGF-inducing capacity of distinct HIF-PHIs differed when evaluated in ARPE-19 cells. It was observed that DMOG and Vadadustat did not significantly affect VEGF secretion in ARPE-19 cells. In contrast, Roxadustat, Molidustat, Enarodustat, and Daprodustat formed a single group and significantly enhanced VEGF secretion to a comparable extent.

It is postulated that the concentration of DMOG applied in the present study may be the reason for the lack of significant increases in VEGF expression, given that other studies have utilized considerably higher doses (100–500 μM) of DMOG in adipose tissue-derived stem cells (ASC) [44] and primary cultures of rat palatal cells [45]. Similar to the results obtained with Vadadustat in ARPE-19 cells, it has been demonstrated that Vadadustat (3–30 μM) does not enhance VEGF secretion when compared to the 1% hypoxia control in Hep 3B cells [46]. In light of our findings, it may be necessary to employ higher doses of DMOG and Vadadustat to achieve a significant increase in VEGF expression, a conclusion that is consistent with the available literature data.

In the concentration range of 5–25 μM, it has been observed that Roxadustat increased VEGF expression in human umbilical vein endothelial cells (HUVECs) [47]. Conversely, Molidustat has showed minimal VEGF secretion in HUVECs, yet it markedly enhances VEGF secretion in human pluripotent stem cell-derived cardiomyocytes, human cardiac ventricular fibroblasts, and human adipose-derived stem cells [48]. Additionally, Molidustat (10–50 μM) has been demonstrated to stabilize HIF-1α and induce the expression of VEGF in MDA-MB-231 breast cancer cells [49]. In our ARPE-19 model, both Roxadustat and Molidustat were observed to induce VEGF production to a similar extent, suggesting that there is no considerable, cell-specific difference between these HIF-PHIs in their ability to induce VEGF in ARPE-19 cells. Daprodustat has also been reported to increase VEGF production in human aortic smooth muscle cells [50]. The data suggest that there may be variability in the capacity of HIF-PHIs to induce VEGF in specific cell types. In general, it seems that HIF-PHIs may have disparate effects on different cell types, which could make them applicable to specific target cells. Further research is required to elucidate these effects in a cell-type-specific manner.

Angiogenin (ANG) is a potent inducer of blood vessel growth [51]. ANG expression is regulated by HIF-1α in ARPE-19 cells and its level is elevated in a murine model of choroidal neovascularization [52]. ANG has also been found to have a correlation with the pathogenesis of neovascular AMD [53]. The present study revealed a pattern of ANG expression similar to that observed for VEGF expression, indicating that HIF-PHIs may exhibit differential potential to induce angiogenic gene expression in ARPE-19 cells. Although VEGF and other angiogenic factors were upregulated in ARPE-19 cells, no tube formation was observed in HUVECs under our experimental conditions. This finding suggests that the induction of angiogenic signaling alone may be insufficient to drive functional angiogenesis in this model. A limitation of our approach is that HUVECs do not fully replicate the morphological or functional characteristics of retinal endothelial cells. Consequently, the applicability of tube formation assays using HUVECs to retinal-specific vascular phenomena is limited. The differential responsiveness of retinal endothelial cells to local angiogenic cues may not be adequately captured in this model, thereby reducing the relevance of these findings to retinal angiogenesis.

GLUT1, which is encoded by Slc2a1, is known to be the primary glucose transporter in the retina pigment epithelium (RPE) [54]. The transport of glucose through the RPE layer via the GLUT1 glucose transporter plays a pivotal role in retinal homeostasis [55]. Notably, HIF-1α has been shown to enhance glycolysis under hypoxic conditions, resulting in increased glucose consumption in the RPE layer, which has been associated with photoreceptor degeneration [41,56]. Here we showed that HIF-PHIs, such as Molidustat, Roxadustat, and Daprodustat, induced GLUT1 expression in ARPE-19 cells at all concentrations tested. In contrast, Vadadustat did not significantly alter GLUT1 levels. It is noteworthy that DMOG and Vadadustat demonstrated comparable gene expression patterns with regard to VEGF and ANG, although they exhibited some differences in terms of GLUT1 induction. It is interesting to observe that the highest dose of DMOG resulted in GLUT1 induction similar to that of Molidustat, Roxadustat, and Enarodustat, suggesting that different HIF-PHIs might induce distinct gene expression patterns. However, in vivo data on how HIF-PHIs can affect GLUT-1 expression are scarce. In a recent study, Roxadustat has been shown to increase GLUT1 expression after 2-week treatment; however, this was not statistically significant compared to the control [34]. Interestingly, GLUT1 protein is significantly reduced in retinas from 4-week Roxadustat-treated mice compared with 2-week mice and GLUT1 protein in 4-week Roxadustat-treated mouse retina is not different from the control group [34]. However, the authors of the study noted that their protein analysis utilized whole retinal lysate, which may have diluted potential response variations across different cells [34]. Here, we showed that acute (16 h) exposure of ARPE-19s to certain HIF-PHIs induced a significant increase in GLUT1 expression. However, it is important to conduct further research to investigate the long-term effects of HIF-PHIs on GLUT1 expression in the eye after prolonged exposure.

In addition to stimulating glycolysis, it has been observed that HIF inhibits the tricarboxylic acid cycle (TCA) by inducing PDK1. This enzyme inactivates the TCA cycle enzyme pyruvate dehydrogenase (PDH), which is responsible for converting pyruvate to acetyl-CoA [57]. PDK1 has been identified as a potential therapeutic target in DR and ROP [58] as well as in neovascular AMD [59]. We observed that Molidustat, Roxadustat, Enarodustat, and Daprodustat significantly induced PDK1 expression in ARPE-19 cells in all tested doses. Similar to our results, Roxadustat significantly elevated PDK1 levels in mice after 4 weeks [34]. We showed that Vadadustat did not induce PDK1 in ARPE-19 cells significantly, while DMOG provoked significant PDK1 induction only at the highest dose. These data indicate that increased PDK1 expression might be a potential off-target effect of HIF-PHIs in the RPE layer, and the effect of long-term HIF-PHI treatment on PDK-1 expression should be investigated in future studies.

Detailed unbiased NGS data on the effect of HIF-PHIs are scarce. To our knowledge, this is the first study that analyses how HIF-PHIs affect RNA transcriptome in ARPE-19s. Our study represents a step forward in our understanding of how HIF-PHIs impact the RNA transcriptome in ARPE-19 cells. Our analysis identified a global RNA transcriptome consisting of 469 DEGs that were significantly altered in response to Roxadustat. The functional characterization of these changes has the potential to uncover both the advantages and disadvantages of HIF-PHIs in retinal pathologies. We found that overrepresented GO terms in Roxadustat-stimulated cells were mostly associated with nucleoside diphosphate metabolic processes, tube development, carbohydrate metabolic processes, methylglyoxal metabolic processes, response to decreased oxygen levels, positive regulation of ATP metabolic processes, neuron death, muscle tissue development, angiogenesis involved in wound healing, regulation of progesterone biosynthetic processes, fructose metabolic processes, and oxidoreductase activity, acting on the aldehyde or oxo group of donors. The gene expression profile of Roxadustat-treated RPEs showed overlap with vitreous markers reported in patients with DR and AMD, as well as in animal models of retinopathy. While this overlap may suggest potential relevance to hypoxia-related pathways implicated in these conditions, further phenotypic and functional studies are needed to establish any pathogenic significance. We showed a significant increase in Roxadustat-treated ARPE-19 cells whose gene expression pattern overlaps with several angiogenic genes involved in DR and AMD, such as ANGPTL4 [60], VEGF [22,23], ANG [52], ADM [61], RORA [62], SERPINE1 [63], ITGB3 [64], ADAMTS1 [65], CXCR4 [66], and LOXL2 [67]. In contrast, other markers of neovascular retinopathy such as CCL2 [68], ANGPTL2 [69], and ID1 [70] were downregulated in the Roxadustat group. Other genes involved in the response to decreased oxygen levels are also associated with retinopathies. Among them, EIF4EBP1, which is upregulated in the retina of diabetic mice [71] and contributes to diabetes-induced vascular dysfunction [72], ALKBH5, which is upregulated in AMD and associated with increased VEGF secretion in RPEs [73], and early growth response 1 (EGR1) transcription factor, which promotes neovascularization in ROP models [74], were significantly induced in ARPE-19 cells exposed to Roxadustat. The overrepresentation of genes (ADM, EGR1, and PPARGC1A)—genes annotated under the ‘regulation of progesterone biosynthetic process’ GO term—suggests that RPE cells may be involved in modulating local steroidogenic pathways. Given the neuroprotective and anti-inflammatory roles of progesterone in the retina [75,76], this transcriptional signature may reflect an adaptive response to cellular stress, mitochondrial demand, or a need for local hormonal signaling.

These findings indicate that a number of genes associated with DR/AMD are upregulated in ARPE-19 cells in response to Roxadustat treatment. While this suggests activation of pathways relevant to retinal disease, it remains unclear whether such changes translate into a DR/AMD-like phenotype, and further phenotypic and functional validation is required.

In general, HIF stabilization induces a glycolytic shift of the TCA cycle toward anaerobic pathways with the upregulation of GLUT1, phosphoglycerate kinase 1 (PGK1), pyruvate dehydrogenase kinase 1 (PDK1), lactate dehydrogenase A (LDHA), hexokinase 2 (HK2), and enolase 1 (ENO1) [77]. Our results show that HIF stabilization by Roxadustat significantly upregulates these glycolytic enzymes in ARPE-19 cells. Energy metabolism in RPE cells appears to play a fundamental role in photoreceptor function and death. Increased glycolysis leading to more robust glucose consumption in the ARPE-19 layer induces photoreceptor degeneration [41,56]. Together, our results indicate that Roxadustat induces a glycolytic shift in RPE cells. Therefore, the potential effects of Roxadustat and other HIF-PHIs on retinal health warrant careful monitoring during long-term systemic administration.

Hypoxia and oxidative stress have been identified as significant factors in the development of age-related macular degeneration (AMD) [78] and diabetic retinopathy (DR) [79]. In addition, hypoxic response to CoCl_2_ is associated with the formation of reactive oxygen species (ROS) in retinal pigment epithelial cells [80]. Here, we showed that Roxadustat did not induce ROS generation in ARPE-19 cells. Although Roxadustat significantly affected the expression of many genes involved in antioxidant response, these expressions showed a heterogeneous pattern since certain antioxidant genes were significantly decreased (PRDX1, PRDX3, PRDX6, TXN2, GSTK1, GSS, GSR), while others (CAT, PRDX2, PRDX4, PRDX5, GSTO1, GSTP1, GSTT2, GSTT2B, GPX3, GPX4, HMOX1, TXNRD1, and TXNRD2) increased in response to Roxadustat. In addition, we did not detect increased ROS generation in cells exposed to Roxadustat. These suggest that Roxadustat-driven hypoxic response is presumably not associated with ROS generation in ARPE-19 cells.

It is well established that inflammation is associated with the pathology of DR. Elevated levels of IL-6, IL-8, and MCP-1 in the aqueous humor are associated with the severity of DR [68,81,82,83] and AMD [84]. Diabetic patients, as evidenced by elevated serum levels of TNF-α, exhibit increased overall inflammatory activity compared to non-diabetic patients [85]. Accordingly, we aimed to ascertain whether HIF-PHI Roxadustat exerts an influence on the secretion of IL-6, IL-8, and MCP-1 in ARPE-19 cells. Our findings indicate that Roxadustat alone did not induce an elevation in IL-6, IL-8, and MCP-1 secretion. However, it was observed that Roxadustat significantly reduced cytokine secretion in response to TNF-α. This finding is consistent with recent studies demonstrating that Roxadustat pretreatment significantly alleviates neuroinflammation in vivo and in vitro [86,87]. This effect was attributed to a reduction in NF-κB phosphorylation in response to lipopolysaccharide [87]. In accordance with the aforementioned findings, we demonstrated that Roxadustat pretreatment attenuated TNF-α-induced phosphorylation of NF-κB p65, which may be implicated in the reduction of pro-inflammatory cytokine expression in ARPE-19 cells in response to TNF-α. The results of this study indicate that Roxadustat does not appear to enhance pro-inflammatory cytokine expression but rather decreases TNF-α-induced inflammation in ARPE-19 cells. While the levels in Roxadustat-treated groups are indeed lower than in the TNF-α-only group, they remain elevated compared to untreated controls. The observation that Roxadustat attenuates TNF-α–induced inflammatory marker (IL-6, IL-8, MCP-1) expression, but does not fully restore levels to baseline, suggests that while HIF stabilization has a modulatory effect on inflammatory signaling in RPE cells, it may not be sufficient to completely counteract proinflammatory stimuli such as TNF-α. Similar observations have been made in cardiomyocytes, where activation of HIF-1 via prolyl-4-hydroxylase-2 silencing attenuated—but did not completely eliminate—TNF-α–induced chemokine and adhesion molecule expression [88].

Our ARPE-19 model has several limitations. ARPE-19 cells are a widely used in vitro model of the human retinal pigment epithelium (RPE) due to their accessibility, ease of culture, and partial expression of RPE-specific markers. However, they do not fully recapitulate the structural and functional characteristics of native RPE cells in vivo, particularly in the context of complex retinal diseases such as AMD and DR. These limitations highlight the importance of validating findings using more physiologically relevant systems, such as induced pluripotent stem cell (iPSC)-derived RPE cells in future studies. Despite these constraints, ARPE-19 cells remain a valuable model for preliminary mechanistic studies and hypothesis generation.

Although the current data indicate that HIF-PHI is not associated with an elevated risk of retinal hemorrhage in comparison to ESA, strict follow-up is recommended if the patient reports visual disturbance after initiation of HIF-PHI, especially in patients with high propensity for retinal complications, such as DR [89]. This is supported by a recent case report which reported the exacerbation of retinopathy in a DR patient following HIF-PHI treatment [90]. In light of these considerations and the established role of hyperglycemia in enhancing VEGF secretion in ARPE-19 cells [36], we sought to investigate the impact of elevated glucose levels on Roxadustat-induced VEGF secretion in ARPE-19 cells. The results demonstrated that high glucose did not exacerbate VEGF secretion in ARPE-19 cells in response to Roxadustat. However, given the potential for other cell types, such as Müller cells and astrocytes, to secrete VEGF in the retina, it cannot be excluded that high glucose might contribute to HIF-PHI-induced retinal neovascularization. Therefore, it is essential to monitor retinal neovascularization and hemorrhage in DR patients undergoing HIF-PHI treatment.

## 5. Conclusions

In conclusion, the data presented herein provide evidence that different HIF-PHIs induce distinct expression of angiogenic (VEGF, ANG) and hypoxic (GLUT-1, PDK-1) factors in ARPE-19 cells. The results of this study demonstrate the induction of a HIF-1α-driven hypoxic response and the activation of pathways involved in angiogenesis and carbohydrate metabolism in ARPE-19 cells treated with the most extensively analyzed HIF-PHI drug Roxadustat. While retinal neovascularization represents a potential adverse effect of HIF-PHIs in the treatment of CKD-associated anemia, the secretome of ARPE-19 cells exposed to HIF-PHI Roxadustat did not demonstrate an increased neovascularization potential in endothelial cultures. Furthermore, HIF-PHI Roxadustat did not induce oxidative stress or a pro-inflammatory response in ARPE-19 cells, both of which are significant risk factors for retinal neovascularization. Although hyperglycemia and diabetes can be a potential risk factor for retinal neovascularization, our findings did not indicate an additive or synergistic effect between high glucose and Roxadustat in VEGF secretion in ARPE-19 cells. In conclusion, ophthalmologic follow-up is recommended during the treatment of CKD patients using PHIs to prevent the potential side effect of HIF-PHIs in retinal/choroidal neovascularization.

## Data Availability

Dataset available on request from the authors. Aligned sequencing data have been deposited into the NCBI SRA database under accession no. PRJNA1092586.

## Supplementary Materials

The following supporting information can be downloaded at: https://www.mdpi.com/article/10.3390/cells14141121/s1, Figure S1: Roxadustat does not induce the translocation of NFκB in ARPE-19 cells.; Table S1: Supplementary Table S1. List of gene abbreviations used in the RNA sequencing (RNA-Seq) analysis.

## Abbreviations

The following abbreviations are used in this manuscript:

AMD	Age-related macular degeneration
ANG	Angiogenin
ARPE-19	Adult retinal pigment epithelial cell-19
BSA	Bovin serum albumin
CAT	Catalase
CKD	Chronic kidney disease
CM	Culture medium
DMEM	Dulbecco’s Modified Eagle Medium
DR	Diabetic retinopathy
ELISA	Enzyme-linked immunosorbent assay
Epo	Erythropoietin
FBS	Fetal bovine serum
FC	Fold change
GLUT1	Glucose transporter-1
GPX3	Glutathione peroxidase 3
GPX4	Glutathione peroxidase 4
GPX8	Glutathione peroxidase 8
GSR	Glutathione-disulfide reductase
GSS	Glutathione synthetase
GSTK1	Glutathione S-transferase kappa 1
GSTO1	Glutathione S-transferase omega 1
GSTP1	Glutathione S-transferase pi 1
GSTT2	Glutathione S-transferase theta 2
GSTT2B	Glutathione S-transferase theta 2B
HIF	Hypoxia-inducible factor
HIF	Hypoxia inducible factor
HMOX1	Heme oxygenase 1
HUVEC	Human Umbilical Vein Endothelial Cell
IL-6	Interleukin-6
IL-8	Interleukin-8
LVES	Large VesseI Endothelial Supplement
MCP-1	Monocyte chemoattractant protein-1
NF-κB	Nuclear factor kappa B
PBS	Phosphate-buffered saline
PDK1	Pyruvate dehydrogenase kinase-1
PHD	Prolyl-4-hydroxylase domain
PHI	Prolyl hydroxylase inhibitor
PRDX1	Peroxiredoxin 1
PRDX2	Peroxiredoxin 2
PRDX3	Peroxiredoxin 3
PRDX4	Peroxiredoxin 4
PRDX5	Peroxiredoxin 5
PRDX6	Peroxiredoxin 6
RIPA buffer	Radioimmunoprecipitation buffer
ROP	Retinopathy of prematurity
ROS	Reactive oxygen species
RT-qPCR	Real-time quantitative polymerase chain reaction
SDS-PAGE	Sodium dodecyl-sulfate polyacrylamide gel electrophoresis
TNF-α	Tumor necrosis factor-α
TXN2	Thioredoxin 2
TXNRD1	Thioredoxin reductase 1
TXNRD2	Thioredoxin reductase 2
TXNRD3	Tthioredoxin reductase 3
VEGFA	Vascular endothelial growth factor-A

## References

[1] KDOQI, National Kidney Foundation (2006). KDOQI Clinical Practice Guidelines and Clinical Practice Recommendations for Anemia in Chronic Kidney Disease. Am. J. Kidney Dis..

[2] Levey A.S., Atkins R., Coresh J., Cohen E.P., Collins A.J., Eckardt K.U., Nahas M.E., Jaber B.L., Jadoul M., Levin A. (2007). Chronic kidney disease as a global public health problem: Approaches and initiatives—A position statement from Kidney Disease Improving Global Outcomes. Kidney Int..

[3] Webster A.C., Nagler E.V., Morton R.L., Masson P. (2017). Chronic Kidney Disease. Lancet.

[4] Grunwald J.E., Alexander J., Ying G.S., Maguire M., Daniel E., Whittock-Martin R., Parker C., McWilliams K., Lo J.C., Go A. (2012). Retinopathy and chronic kidney disease in the Chronic Renal Insufficiency Cohort (CRIC) study. Arch. Ophthalmol..

[5] Kofoed-Enevoldsen A., Jensen T., Borch-Johnsen K., Deckert T. (1987). Incidence of retinopathy in type I (insulin-dependent) diabetes: Association with clinical nephropathy. J. Diabet. Complicat..

[6] Lachin J.M., Genuth S., Cleary P., Davis M.D., Nathan D.M. (2000). Retinopathy and nephropathy in patients with type 1 diabetes four years after a trial of intensive therapy. N. Engl. J. Med..

[7] Park Y.H., Shin J.A., Han J.H., Park Y.M., Yim H.W. (2015). The association between chronic kidney disease and diabetic retinopathy: The Korea National Health and Nutrition Examination Survey 2008–2010. PLoS ONE.

[8] Chen Y.J., Yeung L., Sun C.C., Huang C.C., Chen K.S., Lu Y.H. (2018). Age-Related Macular Degeneration in Chronic Kidney Disease: A Meta-Analysis of Observational Studies. Am. J. Nephrol..

[9] Liew G., Mitchell P., Wong T.Y., Iyengar S.K., Wang J.J. (2008). CKD increases the risk of age-related macular degeneration. J. Am. Soc. Nephrol..

[10] Hirota K. (2021). HIF-α Prolyl Hydroxylase Inhibitors and Their Implications for Biomedicine: A Comprehensive Review. Biomedicines.

[11] Bernhardt W.M., Wiesener M.S., Scigalla P., Chou J., Schmieder R.E., Günzler V., Eckardt K.U. (2010). Inhibition of prolyl hydroxylases increases erythropoietin production in ESRD. J. Am. Soc. Nephrol..

[12] Hsieh M.M., Linde N.S., Wynter A., Metzger M., Wong C., Langsetmo I., Lin A., Smith R., Rodgers G.P., Donahue R.E. (2007). HIF prolyl hydroxylase inhibition results in endogenous erythropoietin induction, erythrocytosis, and modest fetal hemoglobin expression in rhesus macaques. Blood.

[13] Haase V.H. (2021). Hypoxia-inducible factor-prolyl hydroxylase inhibitors in the treatment of anemia of chronic kidney disease. Kidney Int. Suppl. (2011).

[14] Zhao H., Li P., Zhang H.L., Jia L. (2023). An updated meta-analysis on the efficacy and safety of hypoxia-inducible factor prolyl hydroxylase inhibitor treatment of anemia in nondialysis-dependent chronic kidney disease. Ren. Fail..

[15] Lad E.M., Hernandez-Boussard T., Morton J.M., Moshfeghi D.M. (2009). Incidence of retinopathy of prematurity in the United States: 1997 through 2005. Am. J. Ophthalmol..

[16] Lee R., Wong T.Y., Sabanayagam C. (2015). Epidemiology of diabetic retinopathy, diabetic macular edema and related vision loss. Eye Vis..

[17] Ho M., Liu D.T., Lam D.S., Jonas J.B. (2016). Retinal vein occlusions, from basics to the latest treatment. Retina.

[18] Li H.Y., Yuan Y., Fu Y.H., Wang Y., Gao X.Y. (2020). Hypoxia-inducible factor-1α: A promising therapeutic target for vasculopathy in diabetic retinopathy. Pharmacol. Res..

[19] Arjamaa O., Nikinmaa M., Salminen A., Kaarniranta K. (2009). Regulatory role of HIF-1alpha in the pathogenesis of age-related macular degeneration (AMD). Ageing Res. Rev..

[20] Vadlapatla R.K., Vadlapudi A.D., Mitra A.K. (2013). Hypoxia-inducible factor-1 (HIF-1): A potential target for intervention in ocular neovascular diseases. Curr. Drug Targets.

[21] Wang X., Wang G., Wang Y. (2009). Intravitreous vascular endothelial growth factor and hypoxia-inducible factor 1a in patients with proliferative diabetic retinopathy. Am. J. Ophthalmol..

[22] Ng E.W., Adamis A.P. (2005). Targeting angiogenesis, the underlying disorder in neovascular age-related macular degeneration. Can. J. Ophthalmol..

[23] Alon T., Hemo I., Itin A., Pe’er J., Stone J., Keshet E. (1995). Vascular endothelial growth factor acts as a survival factor for newly formed retinal vessels and has implications for retinopathy of prematurity. Nat. Med..

[24] Gáll T., Pethő D., Erdélyi K., Egri V., Balla J.G., Nagy A., Nagy A., Póliska S., Gram M., Gábriel R. (2024). Heme: A link between hemorrhage and retinopathy of prematurity progression. Redox Biol..

[25] Capitão M., Soares R. (2016). Angiogenesis and Inflammation Crosstalk in Diabetic Retinopathy. J. Cell. Biochem..

[26] He J., Jia Z., Zhang A., Bai M. (2024). Long-term treatment of chronic kidney disease patients with anemia using hypoxia-inducible factor prolyl hydroxylase inhibitors: Potential concerns. Pediatr. Nephrol..

[27] Egeolu M., Caleon R.L., Manishimwe E., Zabala Z.E., Moazzami B., Gerges A., O’Keefe G.D., Navarrete J., Galindo R.J., McCoy R.G. (2023). Diabetic retinopathy in African-Americans with end-stage kidney disease: A cross-sectional study on prevalence and impact on quality of life. BMJ Open Diabetes Res. Care.

[28] Adamis A.P., Shima D.T., Yeo K.T., Yeo T.K., Brown L.F., Berse B., D’Amore P.A., Folkman J. (1993). Synthesis and secretion of vascular permeability factor/vascular endothelial growth factor by human retinal pigment epithelial cells. Biochem. Biophys. Res. Commun..

[29] Ponnalagu M., Subramani M., Jayadev C., Shetty R., Das D. (2017). Retinal pigment epithelium-secretome: A diabetic retinopathy perspective. Cytokine.

[30] Somasundaran S., Constable I.J., Mellough C.B., Carvalho L.S. (2020). Retinal pigment epithelium and age-related macular degeneration: A review of major disease mechanisms. Clin. Exp. Ophthalmol..

[31] Sonia S.N., George S., Shahi S.R., Ali Z., Abaza A., Jamil A., Gutlapalli S.D., Ali M., Oble M.J.P., Yu A.K. (2023). An Overview of Safety and Efficacy Between Hypoxia-Inducible Factor-Prolyl-Hydroxylase Inhibitors and Erythropoietin-Stimulating Agents in Treating Anemia in Chronic Kidney Disease Patients. Cureus.

[32] Sears J.E., Hoppe G., Ebrahem Q., Anand-Apte B. (2008). Prolyl hydroxylase inhibition during hyperoxia prevents oxygen-induced retinopathy. Proc. Natl. Acad. Sci. USA.

[33] Hoppe G., Yoon S., Gopalan B., Savage A.R., Brown R., Case K., Vasanji A., Chan E.R., Silver R.B., Sears J.E. (2016). Comparative systems pharmacology of HIF stabilization in the prevention of retinopathy of prematurity. Proc. Natl. Acad. Sci. USA.

[34] Nsiah N.Y., Morgan A.B., Donkor N., Inman D.M. (2023). Long-term HIF-1α stabilization reduces respiration, promotes mitophagy, and results in retinal cell death. Sci. Rep..

[35] Pethő D., Hendrik Z., Nagy A., Beke L., Patsalos A., Nagy L., Póliska S., Méhes G., Tóth C., Potor L. (2021). Heme cytotoxicity is the consequence of endoplasmic reticulum stress in atherosclerotic plaque progression. Sci. Rep..

[36] Schindelin J., Arganda-Carreras I., Frise E., Kaynig V., Longair M., Pietzsch T., Preibisch S., Rueden C., Saalfeld S., Schmid B. (2012). Fiji: An open-source platform for biological-image analysis. Nat. Methods.

[37] Carpentier G., Berndt S., Ferratge S., Rasband W., Cuendet M., Uzan G., Albanese P. (2020). Angiogenesis Analyzer for ImageJ—A comparative morphometric analysis of Endothelial Tube Formation Assay and Fibrin Bead Assay. Sci. Rep..

[38] Mammadzada P., Corredoira P.M., André H. (2020). The role of hypoxia-inducible factors in neovascular age-related macular degeneration: A gene therapy perspective. Cell Mol. Life Sci..

[39] Bindea G., Mlecnik B., Hackl H., Charoentong P., Tosolini M., Kirilovsky A., Fridman W.H., Pagès F., Trajanoski Z., Galon J. (2009). ClueGO: A Cytoscape plug-in to decipher functionally grouped gene ontology and pathway annotation networks. Bioinformatics.

[40] Zhang D., Lv F.L., Wang G.H. (2018). Effects of HIF-1α on diabetic retinopathy angiogenesis and VEGF expression. Eur. Rev. Med. Pharmacol. Sci..

[41] Kurihara T., Westenskow P.D., Gantner M.L., Usui Y., Schultz A., Bravo S., Aguilar E., Wittgrove C., Friedlander M., Paris L.P. (2016). Hypoxia-induced metabolic stress in retinal pigment epithelial cells is sufficient to induce photoreceptor degeneration. Elife.

[42] Puddu A., Ravera S., Panfoli I., Bertola N., Maggi D. (2022). High Glucose Impairs Expression and Activation of MerTK in ARPE-19 Cells. Int. J. Mol. Sci..

[43] Rattner A., Williams J., Nathans J. (2019). Roles of HIFs and VEGF in angiogenesis in the retina and brain. J. Clin. Investig..

[44] Zippusch S., Besecke K.F.W., Helms F., Klingenberg M., Lyons A., Behrens P., Haverich A., Wilhelmi M., Ehlert N., Böer U. (2021). Chemically induced hypoxia by dimethyloxalylglycine (DMOG)-loaded nanoporous silica nanoparticles supports endothelial tube formation by sustained VEGF release from adipose tissue-derived stem cells. Regen. Biomater..

[45] Zhu T., Park H.C., Son K.M., Yang H.C. (2015). Effects of dimethyloxalylglycine on wound healing of palatal mucosa in a rat model. BMC Oral Health.

[46] Zuk A., Si Z., Loi S., Bommegowda S., Hoivik D., Danthi S., Molnar G., Csizmadia V., Rabinowitz M. (2022). Preclinical Characterization of Vadadustat (AKB-6548), an Oral Small Molecule Hypoxia-Inducible Factor Prolyl-4-Hydroxylase Inhibitor, for the Potential Treatment of Renal Anemia. J. Pharmacol. Exp. Ther..

[47] Zhu Y., Wang Y., Jia Y., Xu J., Chai Y. (2019). Roxadustat promotes angiogenesis through HIF-1α/VEGF/VEGFR2 signaling and accelerates cutaneous wound healing in diabetic rats. Wound Repair. Regen..

[48] Coyle R.C., Barrs R.W., Richards D.J., Ladd E.P., Menick D.R., Mei Y. (2021). Targeting HIF-α for robust prevascularization of human cardiac organoids. J. Tissue Eng. Regen. Med..

[49] Kachamakova-Trojanowska N., Podkalicka P., Bogacz T., Barwacz S., Józkowicz A., Dulak J., Łoboda A. (2020). HIF-1 stabilization exerts anticancer effects in breast cancer cells in vitro and in vivo. Biochem. Pharmacol..

[50] Tóth A., Csiki D.M., Nagy B.J., Balogh E., Lente G., Ababneh H., Szöőr Á., Jeney V. (2022). Daprodustat Accelerates High Phosphate-Induced Calcification Through the Activation of HIF-1 Signaling. Front. Pharmacol..

[51] Fett J.W., Strydom D.J., Lobb R.R., Alderman E.M., Bethune J.L., Riordan J.F., Vallee B.L. (1985). Isolation and characterization of angiogenin, an angiogenic protein from human carcinoma cells. Biochemistry.

[52] Lai K., Luo C., Zhang X., Ye P., Zhang Y., He J., Yao K. (2016). Regulation of angiogenin expression and epithelial-mesenchymal transition by HIF-1α signaling in hypoxic retinal pigment epithelial cells. Biochim. Biophys. Acta.

[53] Chen K., Xu W., Zheng J., Shen Y., Ma J., Chen Z. (2020). Angiogenin, FGF-α, and IL-36β have higher expression levels in aqueous humor of nAMD patients in comparison to cataract patients. BMC Ophthalmol..

[54] Rizzolo L.J. (1997). Polarity and the development of the outer blood-retinal barrier. Histol. Histopathol..

[55] Swarup A., Samuels I.S., Bell B.A., Han J.Y.S., Du J., Massenzio E., Abel E.D., Boesze-Battaglia K., Peachey N.S., Philp N.J. (2019). Modulating GLUT1 expression in retinal pigment epithelium decreases glucose levels in the retina: Impact on photoreceptors and Müller glial cells. Am. J. Physiol. Cell Physiol..

[56] Zhao C., Yasumura D., Li X., Matthes M., Lloyd M., Nielsen G., Ahern K., Snyder M., Bok D., Dunaief J.L. (2011). mTOR-mediated dedifferentiation of the retinal pigment epithelium initiates photoreceptor degeneration in mice. J. Clin. Investig..

[57] Kim J.W., Tchernyshyov I., Semenza G.L., Dang C.V. (2006). HIF-1-mediated expression of pyruvate dehydrogenase kinase: A metabolic switch required for cellular adaptation to hypoxia. Cell Metab..

[58] Sato K., Mochida S., Tomimoto D., Konuma T., Kiyota N., Tsuda S., Shiga Y., Omodaka K., Nakazawa T. (2020). A pyruvate dehydrogenase kinase inhibitor prevents retinal cell death and improves energy metabolism in rat retinas after ischemia/reperfusion injury. Exp. Eye Res..

[59] Lambert V., Hansen S., Schoumacher M., Lecomte J., Leenders J., Hubert P., Herfs M., Blacher S., Carnet O., Yip C. (2020). Pyruvate dehydrogenase kinase/lactate axis: A therapeutic target for neovascular age-related macular degeneration identified by metabolomics. J. Mol. Med..

[60] Yang X., Cao J., Du Y., Gong Q., Cheng Y., Su G. (2019). Angiopoietin-Like Protein 4 (ANGPTL4) Induces Retinal Pigment Epithelial Barrier Breakdown by Activating Signal Transducer and Activator of Transcription 3 (STAT3): Evidence from ARPE-19 Cells Under Hypoxic Condition and Diabetic Rats. Med. Sci. Monit..

[61] Ito S., Fujisawa K., Sakamoto T., Ishibashi T. (2003). Elevated adrenomedullin in the vitreous of patients with diabetic retinopathy. Ophthalmologica.

[62] Sun Y., Liu C.H., SanGiovanni J.P., Evans L.P., Tian K.T., Zhang B., Stahl A., Pu W.T., Kamenecka T.M., Solt L.A. (2015). Nuclear receptor RORα regulates pathologic retinal angiogenesis by modulating SOCS3-dependent inflammation. Proc. Natl. Acad. Sci. USA.

[63] Basu A., Menicucci G., Maestas J., Das A., McGuire P. (2009). Plasminogen activator inhibitor-1 (PAI-1) facilitates retinal angiogenesis in a model of oxygen-induced retinopathy. Investig. Ophthalmol. Vis. Sci..

[64] Ning A., Cui J., Maberley D., Ma P., Matsubara J. (2008). Expression of integrins in human proliferative diabetic retinopathy membranes. Can. J. Ophthalmol..

[65] Abu El-Asrar A.M., Nawaz M.I., Allegaert E., Siddiquei M.M., Ahmad A., Gikandi P., De Hertogh G., Opdenakker G. (2022). Differential Expression and Localization of ADAMTS Proteinases in Proliferative Diabetic Retinopathy. Molecules.

[66] Crane I.J., Wallace C.A., McKillop-Smith S., Forrester J.V. (2000). CXCR4 receptor expression on human retinal pigment epithelial cells from the blood-retina barrier leads to chemokine secretion and migration in response to stromal cell-derived factor 1 alpha. J. Immunol..

[67] Van Bergen T., Spangler R., Marshall D., Hollanders K., Van de Veire S., Vandewalle E., Moons L., Herman J., Smith V., Stalmans I. (2015). The Role of LOX and LOXL2 in the Pathogenesis of an Experimental Model of Choroidal Neovascularization. Investig. Ophthalmol. Vis. Sci..

[68] Taghavi Y., Hassanshahi G., Kounis N.G., Koniari I., Khorramdelazad H. (2019). Monocyte chemoattractant protein-1 (MCP-1/CCL2) in diabetic retinopathy: Latest evidence and clinical considerations. J. Cell Commun. Signal.

[69] Keles A., Sonmez K., Erol Y.O., Ayyıldız S.N., Ogus E. (2021). Vitreous levels of vascular endothelial growth factor, stromal cell-derived factor-1α, and angiopoietin-like protein 2 in patients with active proliferative diabetic retinopathy. Graefes Arch. Clin. Exp. Ophthalmol..

[70] Wojnarowicz P.M., Lima E.S.R., Ohnaka M., Lee S.B., Chin Y., Kulukian A., Chang S.H., Desai B., Garcia Escolano M., Shah R. (2019). A Small-Molecule Pan-Id Antagonist Inhibits Pathologic Ocular Neovascularization. Cell Rep..

[71] Schrufer T.L., Antonetti D.A., Sonenberg N., Kimball S.R., Gardner T.W., Jefferson L.S. (2010). Ablation of 4E-BP1/2 prevents hyperglycemia-mediated induction of VEGF expression in the rodent retina and in Muller cells in culture. Diabetes.

[72] Miller W.P., Mihailescu M.L., Yang C., Barber A.J., Kimball S.R., Jefferson L.S., Dennis M.D. (2016). The Translational Repressor 4E-BP1 Contributes to Diabetes-Induced Visual Dysfunction. Investig. Ophthalmol. Vis. Sci..

[73] Sun R.X., Zhu H.J., Zhang Y.R., Wang J.N., Wang Y., Cao Q.C., Ji J.D., Jiang C., Yuan S.T., Chen X. (2023). ALKBH5 causes retinal pigment epithelium anomalies and choroidal neovascularization in age-related macular degeneration via the AKT/mTOR pathway. Cell Rep..

[74] Peng N., Zheng M., Song B., Jiao R., Wang W. (2023). Transcription Factor EGR1 Facilitates Neovascularization in Mice with Retinopathy of Prematurity by Regulating the miR-182-5p/EFNA5 Axis. Biochem. Genet..

[75] de Vries M.H., Redegeld F.A., Koster A.S., Noordhoek J., de Haan J.G., Oude Elferink R.P., Jansen P.L. (1989). Hepatic, intestinal and renal transport of 1-naphthol-beta-D-glucuronide in mutant rats with hereditary-conjugated hyperbilirubinemia. Naunyn Schmiedebergs Arch. Pharmacol..

[76] Sánchez-Vallejo V., Benlloch-Navarro S., López-Pedrajas R., Romero F.J., Miranda M. (2015). Neuroprotective actions of progesterone in an in vivo model of retinitis pigmentosa. Pharmacol. Res..

[77] Lee P., Chandel N.S., Simon M.C. (2020). Cellular adaptation to hypoxia through hypoxia inducible factors and beyond. Nat. Rev. Mol. Cell Biol..

[78] Blasiak J., Petrovski G., Veréb Z., Facskó A., Kaarniranta K. (2014). Oxidative stress, hypoxia, and autophagy in the neovascular processes of age-related macular degeneration. Biomed. Res. Int..

[79] Arden G.B., Sivaprasad S. (2011). Hypoxia and oxidative stress in the causation of diabetic retinopathy. Curr. Diabetes Rev..

[80] Cervellati F., Cervellati C., Romani A., Cremonini E., Sticozzi C., Belmonte G., Pessina F., Valacchi G. (2014). Hypoxia induces cell damage via oxidative stress in retinal epithelial cells. Free Radic. Res..

[81] Dong N., Xu B., Chu L., Tang X. (2015). Study of 27 Aqueous Humor Cytokines in Type 2 Diabetic Patients with or without Macular Edema. PLoS ONE.

[82] Funatsu H., Yamashita H., Noma H., Mimura T., Yamashita T., Hori S. (2002). Increased levels of vascular endothelial growth factor and interleukin-6 in the aqueous humor of diabetics with macular edema. Am. J. Ophthalmol..

[83] Song S., Yu X., Zhang P., Dai H. (2020). Increased levels of cytokines in the aqueous humor correlate with the severity of diabetic retinopathy. J. Diabetes Complicat..

[84] Knickelbein J.E., Chan C.C., Sen H.N., Ferris F.L., Nussenblatt R.B. (2015). Inflammatory Mechanisms of Age-related Macular Degeneration. Int. Ophthalmol. Clin..

[85] Gustavsson C., Agardh C.D., Agardh E. (2013). Profile of intraocular tumour necrosis factor-α and interleukin-6 in diabetic subjects with different degrees of diabetic retinopathy. Acta Ophthalmol..

[86] Ruan Q., Geng Y., Zhao M., Zhang H., Cheng X., Zhao T., Yue X., Jiang X., Jiang X., Hou X.Y. (2024). Prolyl hydroxylase inhibitor FG-4592 alleviates neuroinflammation via HIF-1/BNIP3 signaling in microglia. Biomed. Pharmacother..

[87] Yang D.G., Gao Y.Y., Yin Z.Q., Wang X.R., Meng X.S., Zou T.F., Duan Y.J., Chen Y.L., Liao C.Z., Xie Z.L. (2023). Roxadustat alleviates nitroglycerin-induced migraine in mice by regulating HIF-1α/NF-κB/inflammation pathway. Acta Pharmacol. Sin..

[88] Natarajan R., Salloum F.N., Fisher B.J., Ownby E.D., Kukreja R.C., Fowler A.A. (2007). Activation of hypoxia-inducible factor-1 via prolyl-4 hydoxylase-2 gene silencing attenuates acute inflammatory responses in postischemic myocardium. Am. J. Physiol. Heart Circ. Physiol..

[89] Yap D.Y.H., McMahon L.P., Hao C.M., Hu N., Okada H., Suzuki Y., Kim S.G., Lim S.K., Vareesangthip K., Hung C.C. (2021). Recommendations by the Asian Pacific society of nephrology (APSN) on the appropriate use of HIF-PH inhibitors. Nephrol.

[90] Ariyoshi N., Higashijima F., Wakuta M., Ogata T., Ohta M., Kimura K. (2024). Exacerbation of Diabetic Retinopathy following Hypoxia-Inducible Factor-Prolyl Hydroxylase Inhibitor Administration: A Case Report. Case Rep. Ophthalmol..

