# Supplementary material for: Prolyl Hydroxylase Inhibitor-Mediated HIF Activation Drives Transcriptional Reprogramming in Retinal Pigment Epithelium: Relevance to Chronic Kidney Disease
Source: Cells. 2025 Jul 21;14(14):1121. doi: 10.3390/cells14141121 (PMC12293706; doi:10.3390/cells14141121)

Article

Transcriptional Reprogramming of Retinal Pigment Epithelial Cells by HIF Pathway Activation through Prolyl Hydroxylase Inhibition

Tamás Gáll† [1,2], Dávid Pethő† [1,2,3], Annamária Nagy[1,2,3], Szilárd Póliska[4], György Balla[3,5], József Balla[1,2,3]*

[1] Department of Internal Medicine, Division of Nephrology, Faculty of Medicine, University of Debrecen, Debrecen H-4032, Hungary
[2] Kálmán Laki Doctoral School, University of Debrecen, Debrecen H-4032, Hungary
[3] HUN-REN–UD Vascular Biology and Myocardium Pathophysiology Research Group, Hungarian Academy of Sciences, University of Debrecen, Debrecen H-4032, Hungary
[4] Genomic Medicine and Bioinformatic Core Facility, Department of Biochemistry and Molecular Biology, Faculty of Medicine, University of Debrecen, Debrecen H-4032, Hungary
[5] Department of Pediatrics, Faculty of Medicine, University of Debrecen, Debrecen H-4032, Hungary

* Correspondence balla@belklinika.com

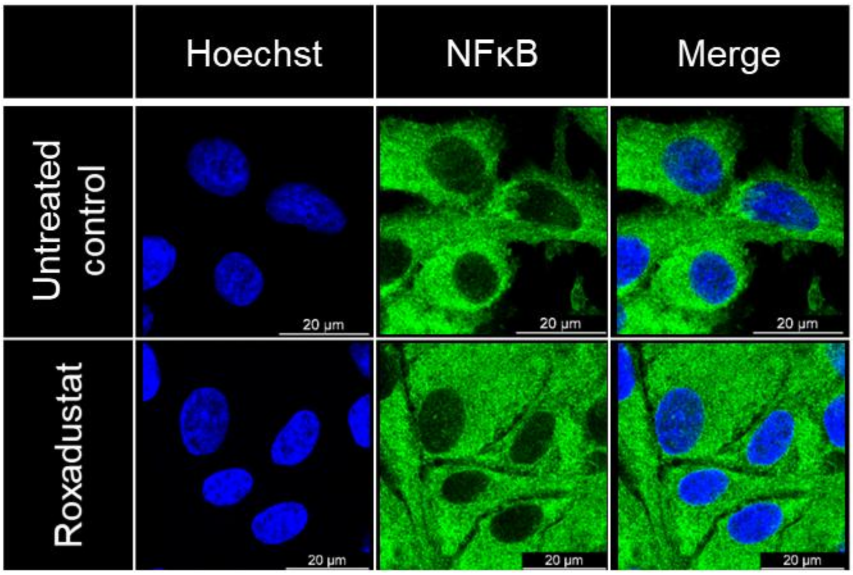

Supplementary figure 1. Roxadustat does not induce the translocation of NFκB in ARPE-19 cells. Cells were exposed to Roxaduxatat (10 µM) and tranlocation of NFκB was analyzed with immunfluorescense. Nuclei were counterstained with Hoechst.

ADAMTS1	ADAM metallopeptidase with thrombospondin type 1 motif 1
ADAMTS9	ADAM metallopeptidase with thrombospondin type 1 motif 9
ADM	adrenomedullin
AK4	adenylate kinase 4
ALKBH5	alkB homolog 5, RNA demethylase
ANG	angiogenin
ANGPTL2	angiopoietin like 2
ANGPTL4	angiopoietin like 4
ARNT2	aryl hydrocarbon receptor nuclear translocator 2
BNIP3	BCL2 interacting protein 3
BNIP3L	BCL2 interacting protein 3 like
CA9	carbonic anhydrase 9
CCL2	C-C motif chemokine ligand 2
CITED2	Cbp/p300 interacting transactivator with Glu/Asp rich carboxy-terminal domain 2
CTGF	connective tissue growth factor
CXCR4	C-X-C motif chemokine receptor 4
CYP1A1	cytochrome P450 family 1 subfamily A member 1
CYP1B1	cytochrome P450 family 1 subfamily B member 1
DDIT4	DNA damage inducible transcript 4
EFNA3	ephrin A3
EGLN1	egl-9 family hypoxia inducible factor 1
EGR1	early growth response 1
EIF4EBP1	eukaryotic translation initiation factor 4E binding protein 1
ENDOG	endonuclease G
ENO1	enolase 1
ERO1A	endoplasmic reticulum oxidoreductase 1 alpha
FAM129B	family with sequence similarity 129 member B
FAM162A	family with sequence similarity 162 member A
FGF18	fibroblast growth factor 18
HILPDA	hypoxia inducible lipid droplet associated
HK2	hexokinase 2
HK2	hexokinase 2
ID1	inhibitor of DNA binding 1, HLH protein
ITGB3	integrin subunit beta 3
KCNK2	potassium two pore domain channel subfamily K member 2
LDHA	lactate dehydrogenase A
LOXL2	lysyl oxidase like 2
MAPK7	mitogen-activated protein kinase 7
NDNF	neuron derived neurotrophic factor
NDRG1	N-myc downstream regulated 1
NFATC4	nuclear factor of activated T-cells 4
NOL3	nucleolar protein 3
NPPB	natriuretic peptide B
PDK1	pyruvate dehydrogenase kinase 1
PDK3	pyruvate dehydrogenase kinase 3
PGK1	phosphoglycerate kinase 1
PLOD2	procollagen-lysine,2-oxoglutarate 5-dioxygenase 2
PPARGC1A	PPARG coactivator 1 alpha
PTGIS	prostaglandin I2 synthase
RORA	RAR related orphan receptor A
SEMA4A	semaphorin 4A

SERPINE1	serpin family E member 1
SFRP1	secreted frizzled related protein 1
SLC2A1	solute carrier family 2 member 1
TGFA	transforming growth factor alpha
TNFAIP3	TNF alpha induced protein 3
VASN	vasorin
VEGFA	vascular endothelial growth factor A

Supplementary Table 1. List of gene abbreviations used in the RNA sequencing (RNA-Seq) analysis. This table provides the full gene names corresponding to the abbreviations used in the RNA-Seq data.

Original blots

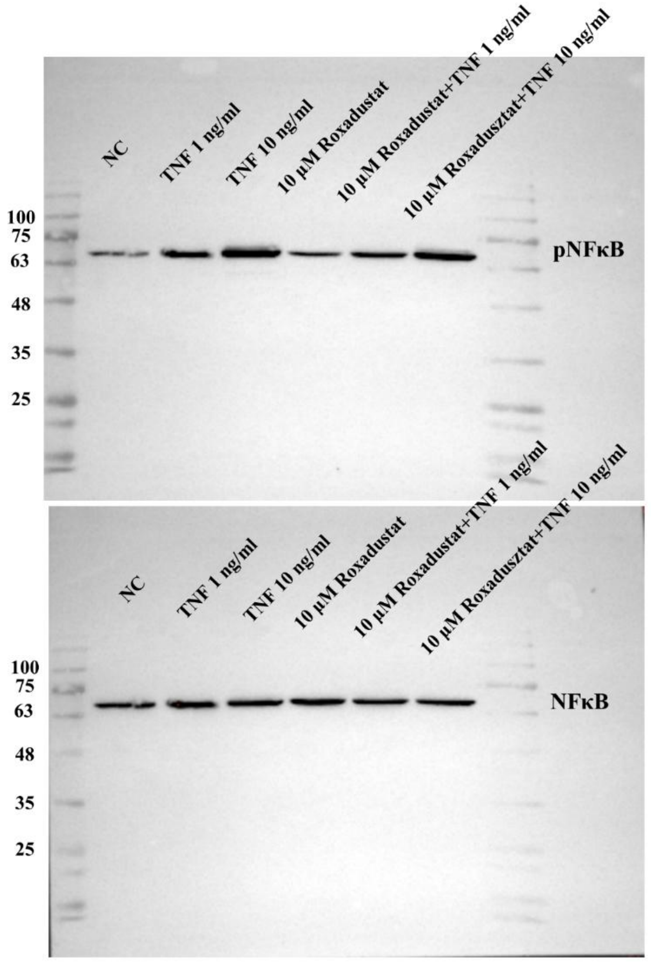

B actin (pNFκB/NFκB)

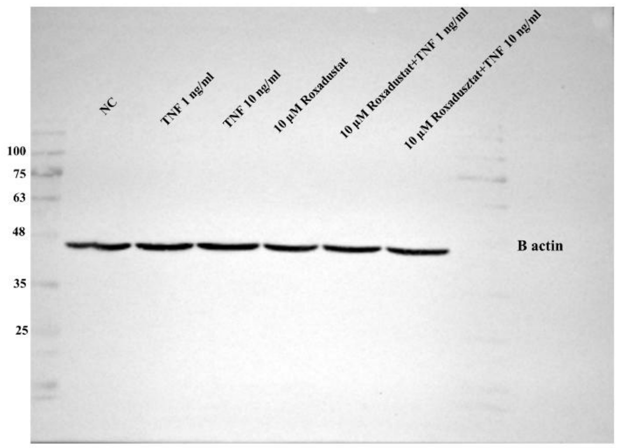

Supplement: Supplementary file 1 [file cells-14-01121-s001.zip › cells-3736321-supplementary.pdf]
